# 6-Amidopyrene as a label-assisted laser desorption/ionization (LA-LDI) enhancing tag: development of photoaffinity pyrene derivative

**DOI:** 10.1038/srep17853

**Published:** 2015-12-15

**Authors:** Kozo Yoneda, Yaping Hu, Masaki Kita, Hideo Kigoshi

**Affiliations:** 1 Graduate School of Pure and Applied Sciences, University of Tsukuba; 2 PRESTO, JST, 1-1-1 Tennodai, Tsukuba 305-8571, Japan

## Abstract

Pyrene-conjugated compounds are detected by label-assisted laser desorption/ionization mass spectrometry (LA-LDI MS) without matrixes. We found that 6-amidopyrene derivatives were highly detectable by the LDI MS instrument equipped with a 355 nm laser. In a certain case of a 6-amidopyrene derivative, a molecular ion peak [M]^+•^ and a characteristic fragment ion peak [M–42]^+•^ were detected in an amount of only 10 fmol. The latter peak, corresponding to the 6-aminopyrene fragment, might be generated *in situ* by the removal of ketene (CH_2_=C=O) from the parent molecule. A photoaffinity amidopyrene derivative of an antitumor macrolide aplyronine A (ApA–PaP) was synthesized, which showed potent cytotoxicity and actin-depolymerizing activity. In an LDI MS analysis of the MeOH- and water-adducts of ApA–PaP, oxime N–O bonds as well as amidopyrene *N*-acetyl moieties were preferentially cleaved, and their internal structures were confirmed by MS/MS analysis. Amidopyrene moiety might enhance fragmentation and stabilize the cleaved fragments by intramolecular or intermolecular weak interactions including hydrogen bonding. Our chemical probe methods might contribute to a detailed analysis of binding modes between various ligands and target biomacromolecules that include multiple and weak interactions.

Identification of the targets of bioactive small molecules (referred to as ligands) is essential in the fields of chemical biology and medicinal chemistry^1^,^2^,^3^. The elucidation of protein- (or other biomacromolecule-) ligand interactions at a molecular level should provide insight for the design and development of new pharmacological tools and drug leads. X-ray crystallographic analysis and NMR spectroscopy have been widely used to analyze these interactions. However, due to the weak interactions and thermal instability of the target molecules, and for other reasons, the interactions with many ligands (especially for natural products and their derivatives) have not been fully established.

Another approach to binding mode analysis is through the use of chemical probes, in which the ligands are conjugated with reacting groups (i.e., photoaffinity and alkyl halide tags) and detecting groups^4^,^5^,^6^,^7^. For example, UV_365_ irradiation of diazirine-ligand conjugates gives a reactive carbene species, which forms covalent bonds with target molecules both *in vitro* and *in situ* (living cells)^8^,^9^. Theoretically, carbene can react with all kinds of amino acid residues in proteins. Enzymatic digestion and subsequent peptide-mass fingerprint (PMF) analysis or Edman degradation of labeled peptides can establish the binding positions of ligands. Meanwhile, especially for non-RI (radioisotope) methods, it is often difficult to detect and purify small quantities of labeled peptides from a mixture of unreacted ligands and other degraded products.

To detect digested peptides in amounts on the order of pg ∼ fg, several kinds of MS analyses with soft ionization methods are now available, such as electrospray ionization (ESI)^10^ and matrix-assisted laser desorption/ionization (MALDI)^11^,^12^,^13^. LDI MS without matrixes is another method for the detection of molecular ions of organic and organometallic compounds that are directly irradiated by UV laser. For example, LDI MS analysis of dipeptides containing aromatic amino acids^14^ and polycyclic aromatic hydrocarbons (PAHs)^15^,^16^,^17^,^18^, such as pyrene and anthracene, have been reported. In 2013, Kozmin and co-workers reported that pyrene-conjugated compounds were selectively detected by LDI MS from the mixture of reactants and reagents, and named this method as label-assisted laser desorption/ionization mass spectrometry (LA-LDI MS)^19^. Since then, several examples related to LA-LDI MS have been reported, which used PAHs or rhodamine fluorophores as detection tags^20^,^21^,^22^.

Thus, we have been focused on LA-LDI MS, and considered the use of chemical probes possessing pyrene and diazirine groups (Fig. 1). After the photolabeling and enzymatic digestion of target proteins, pyrene-labeled peptide(s) should be solely detected on LDI MS. If this approach is successful, the purification of labeled peptides could be omitted, and the binding positions of ligands might be analyzed more efficiently. Here we describe the preparation and mass analysis of photoaffinity pyrene derivatives.

## Results and Discussion

Since PMF analysis is generally performed using on the order of μg~ng proteins, the ability to detect pico- or sub-picomolar amounts by LDI MS is desirable in the case of small amounts of photolabeled products. Kozmin and co-workers detected 35 nmol of 1-pyrenebutyric acid (**1**) with an LDI MS instrument equipped with a 355 nm Nd:YAG laser (UltraXtreme-TN, Bruker)^19^. This amount was enough for the selective detection of pyrene derivatives with sufficient S/N (signal-to-noise) ratio from the mixture of unlabeled compounds. With the same instrument, we tried to reduce the amounts of samples, and could detect at least 100 pmol of **1**, while detection was not clear with 10 pmol (Fig. 2). On the UV–Vis absorption spectrum of **1**, the maximum absorbance wavelength was 340 nm, and there was little absorption at 355 nm, which might lead to the low sensitivity on LDI MS.

Several aromatic amines have strong absorbance and/or fluorescence properties under UV_365_ irradiation, such as *p*-nitroaniline and 7-amino-4-methylcoumarine (AMC). In addition, due to their high cationic properties, AMC and Alexa Fluor^®^ 350 dyes were shown to enhance the sensitivity of ESI or MALDI-TOF MS^23^,^24^,^25^. Thus, we initially planned to use an aminopyrene chromophore as an LDI-enhancing tag. 6-Aminopyrene **2** and amidopyrene **3** were prepared from **1** via nitration, reduction, and *N*-acetylation (Scheme S1 and Fig. S1, Supplementary Information). The UV absorbance of both **2** and **3** shifted to a longer wavelength (λ_max_ 362 and 346 nm, respectively), with substantial absorption at 355 nm (Fig. 2a).

In the LDI mass analysis of **2**, a molecular ion peak at *m/z* 317.2 [M]^+•^ was observed as a radical cation, with a base peak at *m/z* 632.3 (Fig. 2b). The latter peak was assigned to be a homodimer (radical) cation [2M–2H]^+•^. Aniline and related aromatic amines are known to dimerize with acidic treatment or photoirradiation to give hydrazines and other compound mixtures^26^, so that **2** was oxidatively-dimerized by UV laser irradiation. Due to the complexity of mass spectra, aminopyrene was thought to be unsuitable for LA-LDI-enhancing tags. In contrast, in the case of amidopyrene **3**, a molecular ion peak at *m/z* 359.2 ([M]^+•^, base peak) and a fragment ion at *m/z* 317.2 [M–42]^+•^ were solely detected with no dimerized ion peaks (Fig. 2b), and the both peaks were detected in amounts of only 10 fmol (Fig. S2, Supplementary Information). Acetanilide is known to fragment by losing ketene (CH_2_=C=O), producing the peak of aniline on EI (electron ionization) MS analysis^27^,^28^,^29^. Thus, the latter peak corresponding to the molecular ion of aminopyrene **2** might be generated *in situ* by the removal of ketene from **3**. Meanwhile, methyl 4-(1-pyrenyl)butyrate were detected by LDI MS in amounts of 10–100 fmol, and the S/N ratios of molecular ion were only 3–5 times lower than those of amidopyrene **3** (Fig. S2, Supplementary Information). These results suggested that the protection of carboxylic acid moiety as well as the presence of amide group in pyrene moiety might improve sensitivity on LDI MS. Notably, **3** was solely detected on LDI MS in the presence of angiotensin I, a tyrosine- and histidine-containing decapeptide that is used as a standard for MALDI-TOF MS (Fig. S3, Supplementary Information).

Next, to validate our new chemical probe method, we chose as a ligand the antitumor marine macrolide aplyronine A (ApA), which shows potent actin-depolymerizing activity (Fig. 3)^30^,^31^,^32^,^33^. We planned to compare the positions of photolabeling sites determined by LDI MS with that of ApA in the actin–ApA complex^34^. It was found that the C34 *N*-methyl enamide moiety of ApA can be replaced with oximes without a significant loss of activity^35^,^36^. Also, several PEG-linked biotin conjugates of aplyronines have been shown to retain the potent activity of the natural products^37^,^38^. Based on these findings, amidopyrene **3** was converted to an alkoxyamine derivative possessing an aryltrifluoromethyldiazirine^39^,^40^ group and a PEG linker, and coupled with ApA C34 aldehyde to afford an aplyronine A photoaffinity amidopyrene derivative (ApA–PaP, **4**) (Schemes S2 & S3, Supplementary Information). As a model experiment for the photolabeling of target proteins, compound **4** was reacted with solvents under irradiation with UV_365_ at 0 °C for 15 min to give the MeOH- and water-adducts of ApA–PaP (**6** and **8**) in 42% yield, respectively. For comparison, ApA photoaffinity pyrene deriveative (ApA–PP, **5**) was also synthesized, and was photoreacted in methanol to afford the MeOH-adduct of ApA–PP (**7**). The planar structures of **6** and **7** were estimated as shown in Fig. 3, since carbenes generated from trifluoromethylaryldiazirines are known to react with MeOH by O–H insertion in preference to C–H insertion^41^. The UV absorbance spectra and relative intensity at 355 nm of the MeOH-adducts of ApA–PaP (**6**) and ApA–PP (**7**) were almost coincided with those of amidopyrene (**3**) and methyl 4-(1-pyrenyl)butyrate, respectively (Figure S4, Supplementary Information).

In the LDI MS analysis of the MeOH-adduct **6**, the molecular ion peak [M + Na]^+^ and a fragment ion [M + Na–CH_2_CO]^+^, were slightly observed in amount of more than 100 pmol (data not shown). Meanwhile, two pairs of characteristic fragment ions with differences of 42 mu were mainly observed at *m/z* 949.4/907.4 and 573.3/531.3 even in an amount of 1 pmol, which were assumed to be cleaved at the oxime N–O bond and the carbonyl α position of a Lys group, respectively (Fig. 4a). Another peak at *m/z* 875.4 was assigned as the fragment cleaved at the ε-amino group. On the same condition, no fragment ions that were generated by the cleavage of the PEG C–O bonds, other amide C–N bonds, or the photoreacted sites were observed. As for the water-adduct **8**, two fragment ions at *m/z* 893.4 and 861.4 were observed in an amount of 10 pmol, which were 14 mu (CH_2_) smaller than those observed in **6**, while the peaks at *m/z* 573.3/531.3 were the same (Fig. S5a, Supplementary Information). These results strongly suggest that the amidopyrene C–N bonds and the oxime N–O bonds were preferentially cleaved to generate aminopyrene fragments *in situ*, regardless of the structures and sizes of the ligands or the adducts of photoreactions.

On the LDI MS, the relative intensity of molecular ion (M^+•^) was almost twice higher than that of the fragment ion ([M – CH_2_CO]^+•^) for amidopyrene **3** between 10 fmol and 50 pmol amounts (see Figs S1 and S2, Supplementary Information). However, when amidopyrene was used as a tag, the parent fragment ions (i.e. *m/z* 949.4 and 573.3 for the MeOH-adduct **6**) were observed far less than their ketene-liberated fragment ions (*m/z* 907.4 and 531.3, respectively) (Fig. 4a). This tendency was almost the same as the case of water-adduct **8**. Also, the power of laser irradiation on LDI MS instrument hardly affected the relative intensities of these fragment ion peaks. Therefore, it was expected that amidopyrene moiety could enhance fragmentation and stabilize the cleaved fragments by intramolecular or intermolecular weak interactions including hydrogen bonding.

In the case of the MeOH-adduct of pyrene derivative **7**, two major fragment ions were observed at *m/z* 915.4 and 491.3 as sodium ion or proton adducts in an amount of 1 pmol, along with several unassignable fragment ions on LDI MS (Fig. 4c). The former ion corresponded to the fragment cleaved at the oxime N–O bond as in the case of **6**. However, the latter ion was assigned as the fragment cleaved at the amide C–N bond of a Lys group, not at the carbonyl α position. These results suggested that, in the cases of amidopyrenes **6** and **8**, their pyrene moieties would have a certain role to stabilize the *N*-carbonyl cations generated *in situ*.

**Figure 1:**
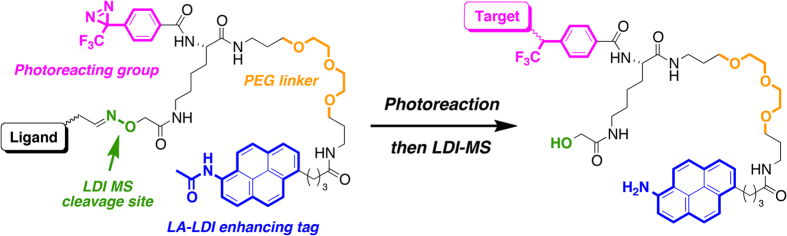
Overall strategy of photoaffinity pyrene probes for LA-LDI MS.

**Figure 2:**
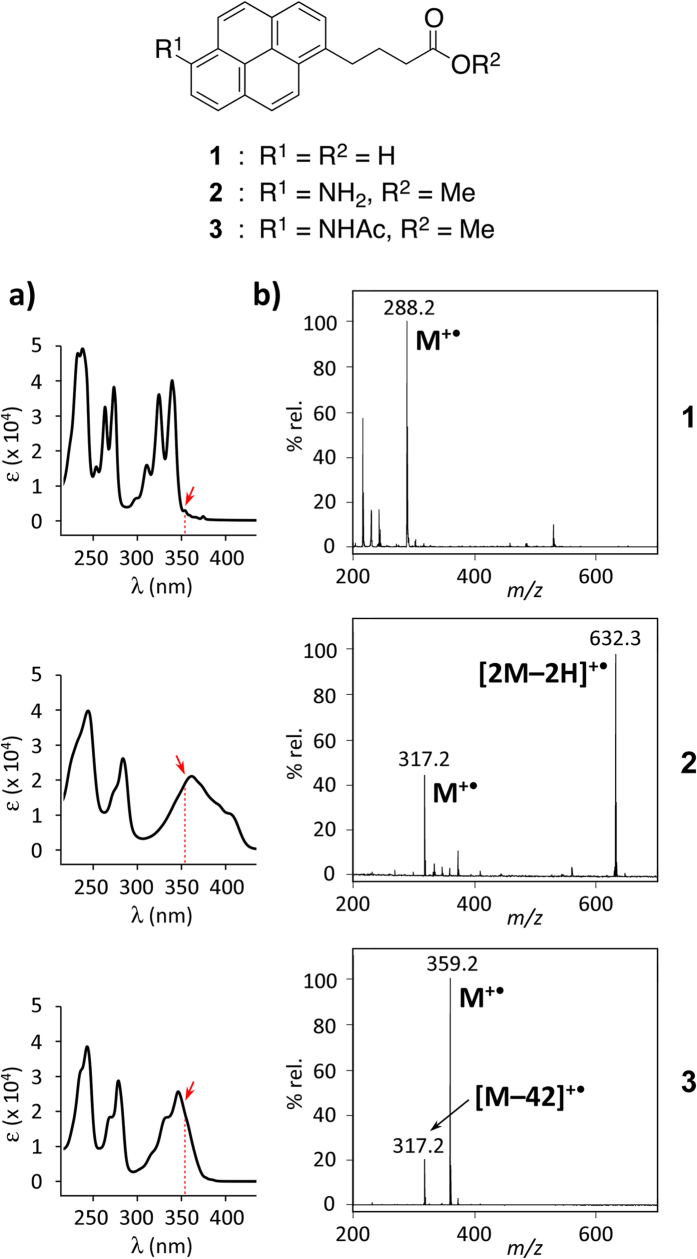
UV absorbance and LDI MS of pyrenes. (**a**) UV–Vis absorption spectra of pyrene derivatives **1**–**3**. A red arrow suggests the absorption at 355 nm. (**b**) LDI mass spectra of **1** (100 pmol) and **2**–**3** (5 pmol each).

**Figure 3:**
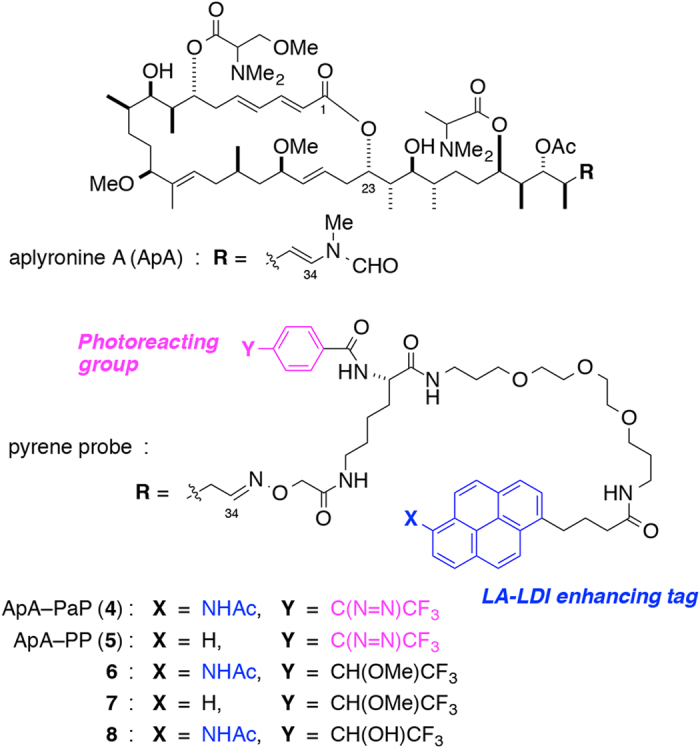
Structures of aplyronine A and its photoaffinity pyrene derivatives.

**Figure 4:**
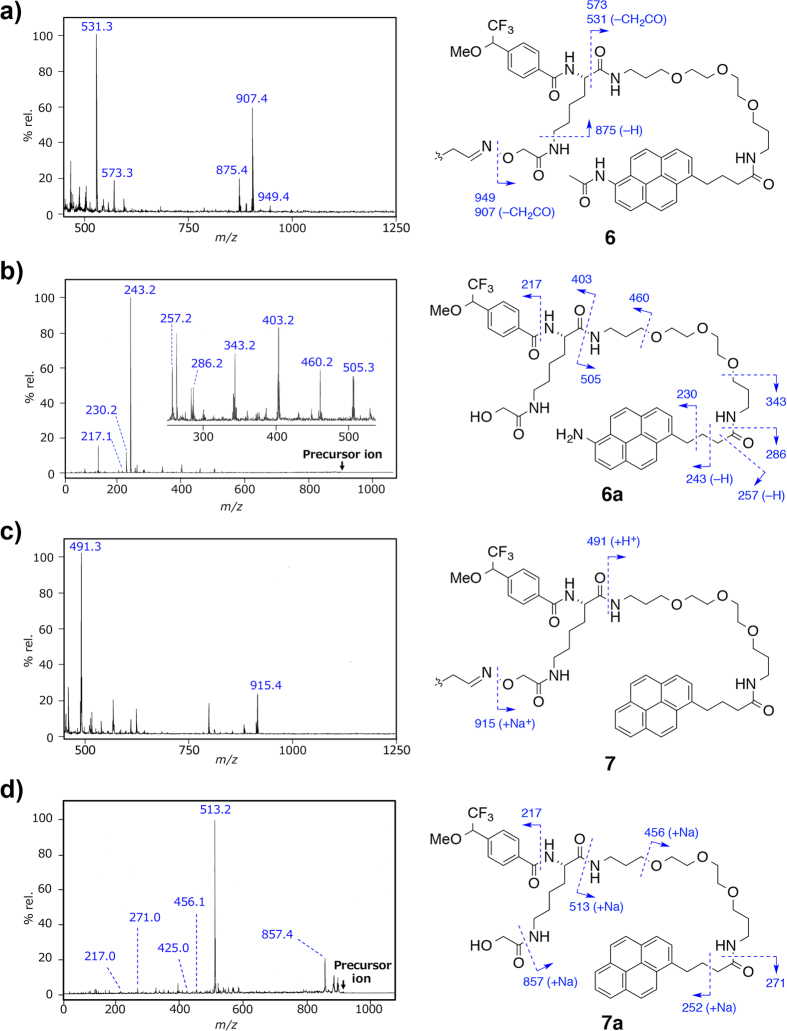
LDI MS and MS/MS analyses. (**a**) LDI mass spectrum of the MeOH-adduct of ApA–PaP (**6**) (1 pmol). (**b**) MS/MS analysis of the fragment **6a** generated from **6** (1pmol). Precursor ion: *m/z* 907.4. (**c**) LDI mass spectrum of the MeOH-adduct of ApA–PP (**7**) (1 pmol). (**d**) MS/MS analysis of the fragment **7a** generated from **7** (1 pmol). Precursor ion: *m/z* 915.4. The fragmentation mass peaks assigned in (**a**) to (**d**) are shown in each chemical structure (right).

Since the aim of this research is to detect the conjugates of targets with amido- or aminopyrene tags, as shown in Fig. 1, it was desirable to detect the fragment ions specifically cleaved at the oxime N–O bonds (i.e. *m/z* 949.4 and 907.4 for **6**). To establish the structure of the major LDI MS fragment **6a** (*m/z* 907.4) generated from **6**, a MS/MS analysis was performed (Fig. 4b). Along with a typical MALDI-TOF MS/MS analysis, the amide C–N bonds and PEG C–O bonds were cleaved, which were surely assigned by comparison with those of **8a** (*m/z* 893.4) generated from **8** (Fig. S5b, Supplementary Information). Among them, two pairs of peaks at *m/z* 403.2/505.3 in **6a** and *m/z* 389.3/505.4 in **8a** were assigned to be the acylium and ammonium fragment ions that were generated by the cleavage at the Lys C-terminus amides, respectively. In contrast, no peaks derived from the cleavage of carbonyl α position of a Lys group were observed. In both MS/MS analyses, most of the fragment ions were observed at the pyrene-containing site, probably due to the positive charge of aminopyrene groups. Notably, a fragment ion at *m/z* 243.2 was predominantly observed in both MS/MS analyses, and was assigned to be a 6-amino-1-vinylpyrene cation formed by McLafferty rearrangement at the 1-pyrenebutylamide moiety. This characteristic MS/MS fragment ion peak is likely to be a suitable marker for the detection of pyrene-containing molecules in digested peptide mixtures.

For comparison, MS/MS analysis of the pyrene fragment **7a** (*m/z* 915.4) generated from **7** were conducted (Fig. 4d). Four ion peaks at *m/z* 857.4, 513.2 (base peak), 271.0, and 217.0 were assigned as the fragments cleaved at the amide C–N bonds. However, compared with the aminopyrene fragments **6a** and **8a**, the pyrene fragment **7a** had more complex fragmentation pattern on MS/MS analysis. These differences in fragmentation patterns could be explained by the presence of amino group in aminopyrenes to facilitate effective charge remote fragmentation.

To address the difference in the sensitivity and fragmentation patterns between 6-amidopyrene and known pyrene compounds on LDI MS, we considered the weak interactions such as hydrogen bonding on the excited states. It has been shown that electronic excited-state hydrogen bonding dynamics have important roles on internal conversion, electronic spectral shifts, photoinduced electron transfer (PET), intramolecular charge transfer (ICT), and so on^42^. For example, the fluorescence quenching of the oxazine 750 dye in protic solvents is caused by the solute–solvent intermolecular PET from protic alcohols to the chromophore via intermolecular hydrogen bonds^43^. In fact, in comparison with the almost same fluorescence of pyrene **9** in acetonitrile and MeOH, significant fluorescence quenching for amidopyrene **3** was observed in the protic solvent (~50% reduce) (Fig. S6, Supplementary Information). Thus, even under the highly vacuum and solid conditions, intra- or intermolecular hydrogen bonds could facilitate the PET or ICT of amidopyrene moieties, which might contribute to the unique fragmentation and stabilization of the fragments possessing amidopyrene (and aminopyrene generated *in situ*) tags on the LDI MS.

**Figure 5:**
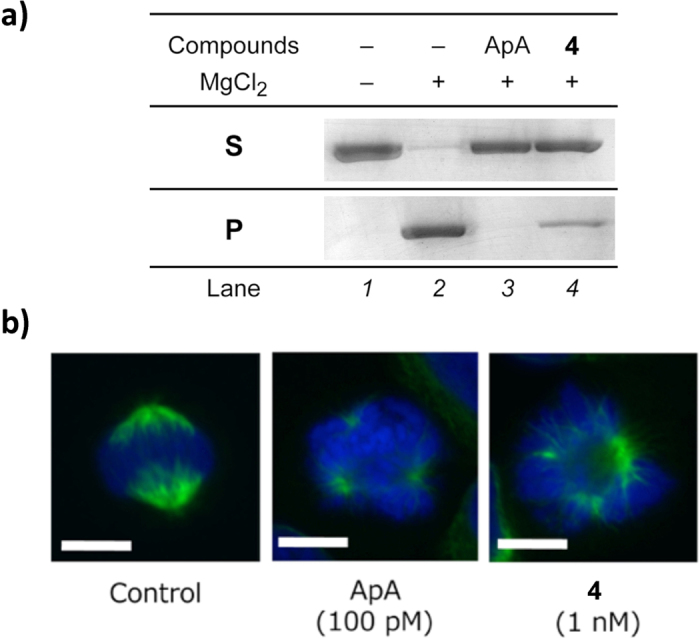
Biological activity of ApA–PaP. **(a**) *In vitro* F-actin sedimentation assay. After treatment with ApA (5 μM) and **4** (50 μM), filamentous (F-) actin (3 μM as a monomer) was precipitated by ultracentrifugation. Proteins contained in the supernatant (**S**) and the precipitate (**P**) were analyzed by SDS-PAGE. (**b**) Confocal fluorescence images of HeLa S3 cells (metaphase) treated with ApA or probe **4** for 6 h. Cells were immunostained with anti-α-tubulin (green) and costained with DAPI (blue). Scale bar, 10 μm.

With respect to the biological activity, ApA–PaP (**4**) showed potent cytotoxicity against HeLa S3, a human cervical carcinoma cell line (IC_50_ 0.66 nM). An *in vitro* F-actin sedimentation assay^36^ showed that actin polymerization was almost completely inhibited by treatment with **4** (17 eq. for 3 μM actin, *lane 4*), as with that of ApA (1.7 eq., *lane 3*) (Fig. 5a). Recently, aplyronine A was shown to induce protein–protein interaction between actin and tubulin to prevent spindle formation and mitosis in tumor cells^44^,^45^. In fact, HeLa S3 cells treated with 1 nM ApA–PaP (**4**) had abnormal multipolar spindles, as in the case of 100 pM ApA (Fig. 5b). These results suggested that the amidopyrene group with a hydrophilic PEG linker had little effect on the bioactivity of ApA.

In summary, we demonstrated that 6-amidopyrene was an efficient LA-LDI enhancing tag for mass analysis. A highly bioactive aplyronine A photoaffinity amidopyrene derivative was synthesized, and LDI MS and MS/MS analyses of its photoreacted products established their fragment structures. To our knowledge, this is the first example of the use of amidopyrene derivatives as photoaffinity probes and the first demonstration of their outstanding potential in mass analyses. Our methods may be useful for the analysis of binding properties between various ligands and target molecules that include multiple or weak interactions. Further studies on LDI MS-applicable chemical probes, including the efficient photolabeling of target proteins and the detection of labeled peptides at sub-picomol amounts, are currently underway.

## Methods

### Chemical synthesis

Information in detail was provided in Supporting Information.

### LDI MS analysis

Matrix-assisted laser desorption/ionization with time-of-flight mass spectrometry (MALDI-TOF MS) was performed using a Bruker UltrafleXtreme spectrometer, equipped with a 355 nm Nd:YAG laser, with α-cyano-4-hydroxycinnamic acid as a matrix. Label-assisted laser desorption/ionization mass spectrometry (LA-LDI MS) was performed using the same apparatus as for MALDI-TOF MS. Samples dissolved in 50% aq. MeOH or MeCN/1% TFA were spotted to an MTP384 ground steel target plate and air-dried according to the manufacturer’s instructions.

### Photolabeling experiments

To protect aryl diazirine derivatives from light, all experiments were conducted with a light-shaded plastic tube (0.6 mL). An aplyronine photoaffinity derivative, 2 mM ApA–PaP (**4**) in DMSO (1 μL), was dissolved in MeOH or water (100 μL). The solutions were cooled on ice and irradiated with UV light (365 nm) for 15 min, using a handheld UV lamp (0.8 mW/cm^2^). The reaction mixture was concentrated *in vacuo* and applied to a Develosil ODS-HG-5 HPLC column (ϕ 4.6 mm I.D. × 250 mm). Samples were eluted with MeOH/20 mM ammonium acetate (81:19) at a flow rate of 1 mL/min and with monitoring by fluorescence (λ_ex_ 337 nm and λ_em_ 409 nm) to give the MeOH-adduct **6** and the water-adduct **8** in 42% yield each (based on the fluorescence in HPLC analysis). Similarly, the MeOH-adduct **7** was prepared from ApA–PP (**5**) in 8% yield.

### *In vitro* actin-depolymerizing activity assay

The actin-depolymerizing activities were measured based on F-actin sedimentation (centrifugation method), as previously described^36^. A 0.15 M solution of MgCl_2_ (3.3 μL) was added to a solution of rabbit muscle actin (2 μM, Cytoskeleton) in G-buffer (500 μL), and the mixture was stirred at 25 °C for 1 h and then ultracentrifuged (150,000 × *g*, 22 °C, 1 h). The precipitate was resuspended in G-buffer including 1 mM MgCl_2_ to give an F-actin solution. The protein concentration was measured with a Bio-Rad Protein Assay Kit (Bradford’s method) with BSA as a standard. Samples were added to the solutions of F-actin (0.50 mL), and the resulting mixtures were stirred at 25 °C for 1 h and then ultracentrifuged (150,000 × *g*, 22 °C, 1 h). The supernatants (lyophilized) and the precipitates were dissolved in 1× SDS buffer (100 μL, Sigma) and boiled at 95 °C for 5 min. SDS-PAGE was performed using a precast 10% polyacrylamide gel (ATTO), and the gels were stained with a Quick-CBB kit (Wako).

### Cell culture and cytotoxicity assay

HeLa S3 cells (suspension culture-adapted human cervical carcinoma cell line, ATCC CCL-2.2) were cultured in Eagle’s minimal essential medium (E-MEM) supplemented with fetal bovine serum (FBS, 10%) in a humidified atmosphere containing CO_2_ (5%). For bioassays, ApA and its derivatives were stored in DMSO at 1 mM, unless otherwise noted. The cytotoxicity of ApA and its derivatives were measured by the 3-(4,5-dimethylthiazol-2-yl)-2,5-diphenyltetrazolium bromide (MTT) method. HeLa S3 cells were seeded at 2 × 10^3 ^cells per well in 96-well plates. After cells were incubated overnight at 37 °C, aplyronines (1 pM–1 μM) were added, and cells were incubated for an additional 96 h at 37 °C. A 1.4 mg/mL MTT solution in phosphate buffer saline (PBS) (50 μL) was added to the cells. After 4 h, the culture medium was removed and the formazan product was dissolved in DMSO (150 μL). Optical density at 540 nm was measured with a TECAN microplate reader (Infinite^®^ 200 Pro). All assays were performed in duplicate to confirm reproducibility.

### Immunofluorescence staining

Exponentially growing HeLa S3 cells were seeded on an 8-well glass chamber slide coated with collagen (Lab-Tek™, Nunc) at 1.2 × 10^4^ cells per 0.25 mL. The cells were incubated for 24 h at 37 °C prior to the addition of samples. Cells that had been treated with samples for 6 h at 37 °C were washed with PBS and fixed with MeOH (250 μL) for 30 min at −20 °C. After being washed with PBS, the cells were blocked with 0.5% BSA in PBS for 1 h at room temperature. The cells were then incubated in anti-α-tubulin monoclonal antibody DM1A (cat. no. sc-32293, Santa Cruz Biotechnology) at 0.8 μg/mL, diluted in the blocking buffer, for 1 h at room temperature. The cells were washed with 0.1% BSA/PBS and incubated in Alexa Fluor^®^ 488 anti-mouse IgG (Invitrogen) at 2 μg/mL, diluted in 0.1% BSA/PBS. The mixture was left to stand for 1 h at room temperature. After being washed four times with PBS, 4′,6-diamidino-2-phenylindole (DAPI, DOJINDO) at 0.5 μg/mL in PBS was added, and the mixture was left to stand for 1 h. The cells were again washed with PBS, the polystyrene chambers were removed, and slides were mounted with SlowFade^®^ Gold antifade reagent (Invitrogen). Fluorescence and bright-field images of fixed cells were captured using an Olympus FV1000-D laser scanning confocal microscope.

## Additional Information

**How to cite this article**: Yoneda, K. *et al.* 6-Amidopyrene as a label-assisted laser desorption/ionization (LA-LDI) enhancing tag: development of photoaffinity pyrene derivative. *Sci. Rep.* **5**, 17853; doi: 10.1038/srep17853 (2015).

## Supplementary Material

Supplementary Information
