# Supplementary material for: 6-Amidopyrene as a label-assisted laser desorption/ionization (LA-LDI) enhancing tag: development of photoaffinity pyrene derivative
Source: Sci Rep. 2015 Dec 15;5:17853. doi: 10.1038/srep17853 (PMC4678867; doi:10.1038/srep17853)
Supplement: Supplementary Information [file srep17853-s1.pdf]

Supporting Information for

6-Amidopyrene as a label-assisted laser desorption/ionization (LA-LDI) enhancing tag: development of photoaffinity pyrene derivative

Kozo Yoneda,[1] Yaping Hu,[1] Masaki Kita*[1,2] & Hideo Kigoshi*[1]

[1]*Graduate School of Pure and Applied Sciences, University of Tsukuba, and* [2]*PRESTO, JST, 1-1-1 Tennodai, Tsukuba, Ibaraki 305-8571, Japan*

(27 pages)

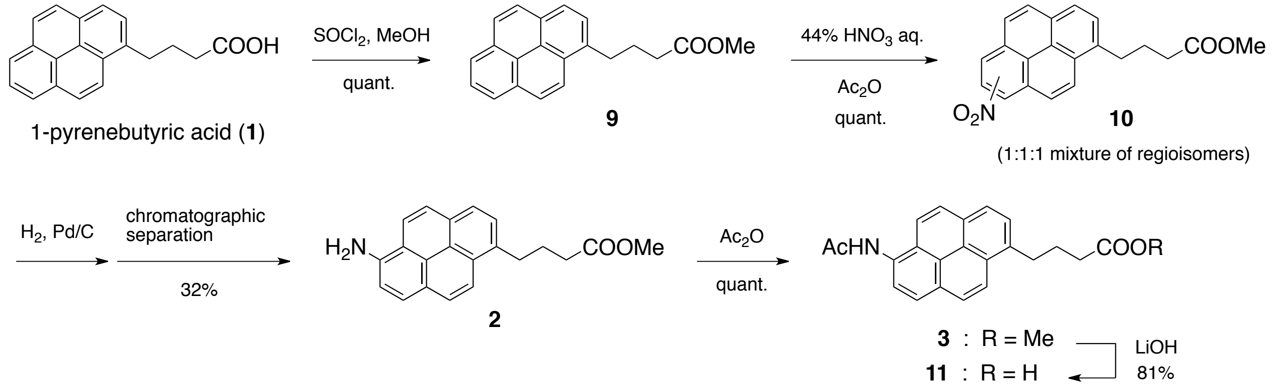

Scheme S1. Synthesis of amidopyrene carboxylic acid **11**.

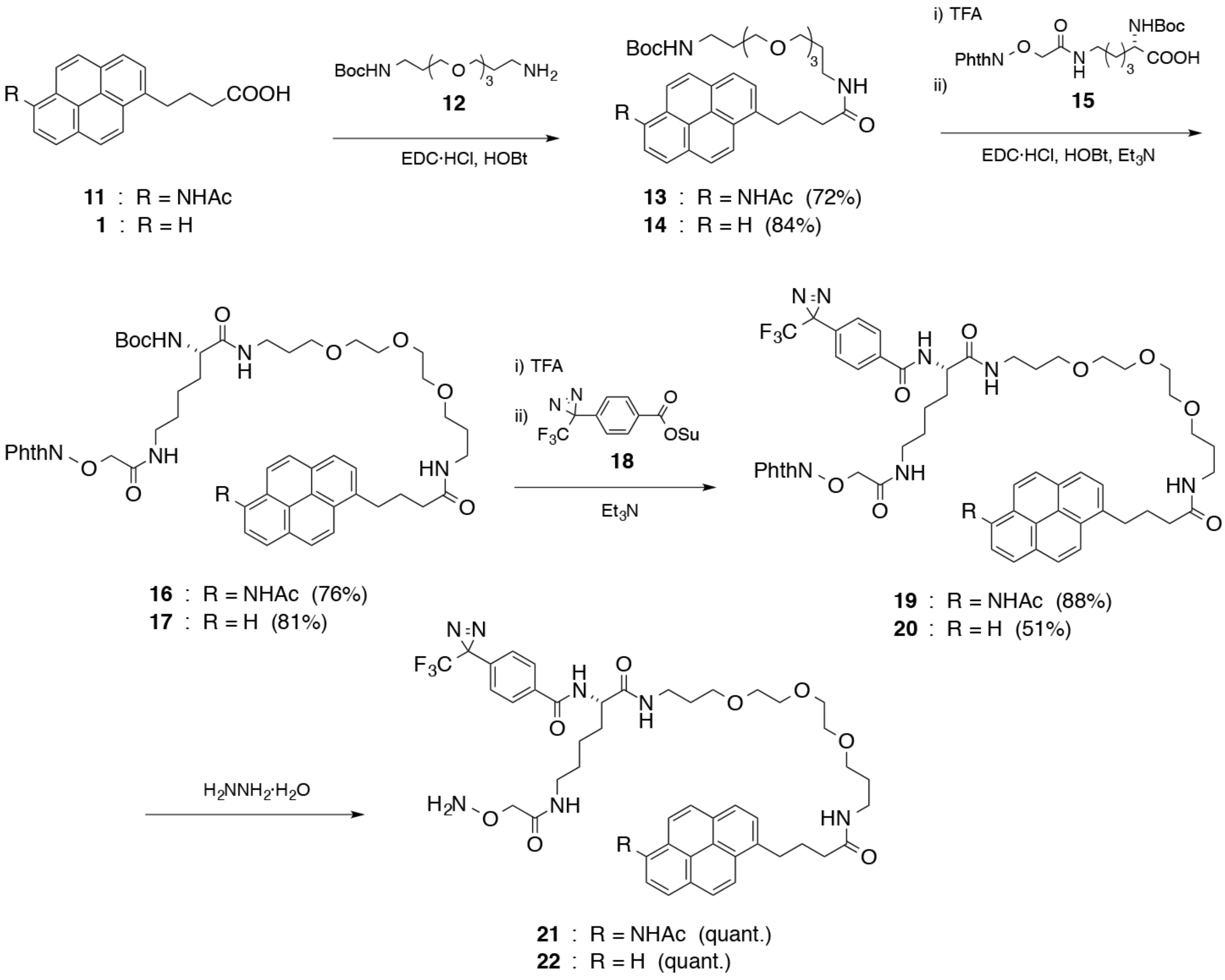

Scheme S2. Synthesis of alkoxyamines **21** and **22**.

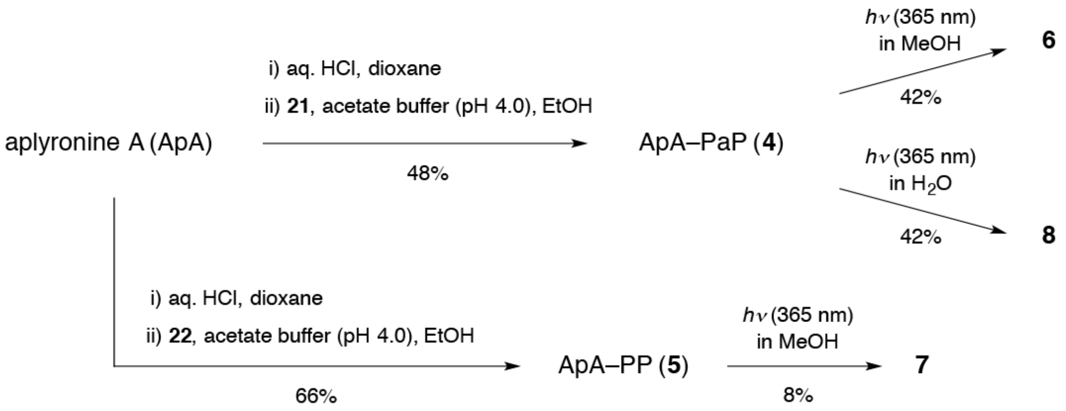

Scheme S3. Synthesis of ApA photoaffinity pyrene derivatives **4** and **5** and their model photoreactions.

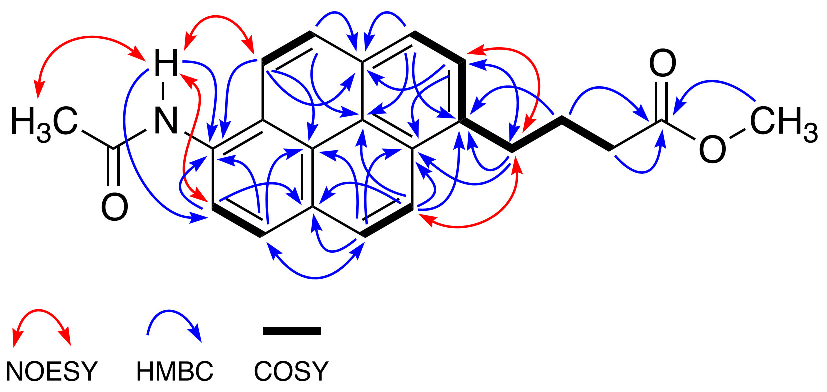

Figure S1. 2D-NMR analysis of amidopyrene **3**. The position of *N*-acetyl group in **3** was established by 2D-NMR analysis.

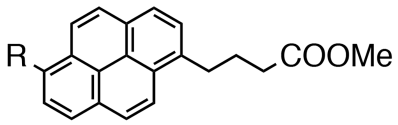

3 : R = NHAc
9 : R = H

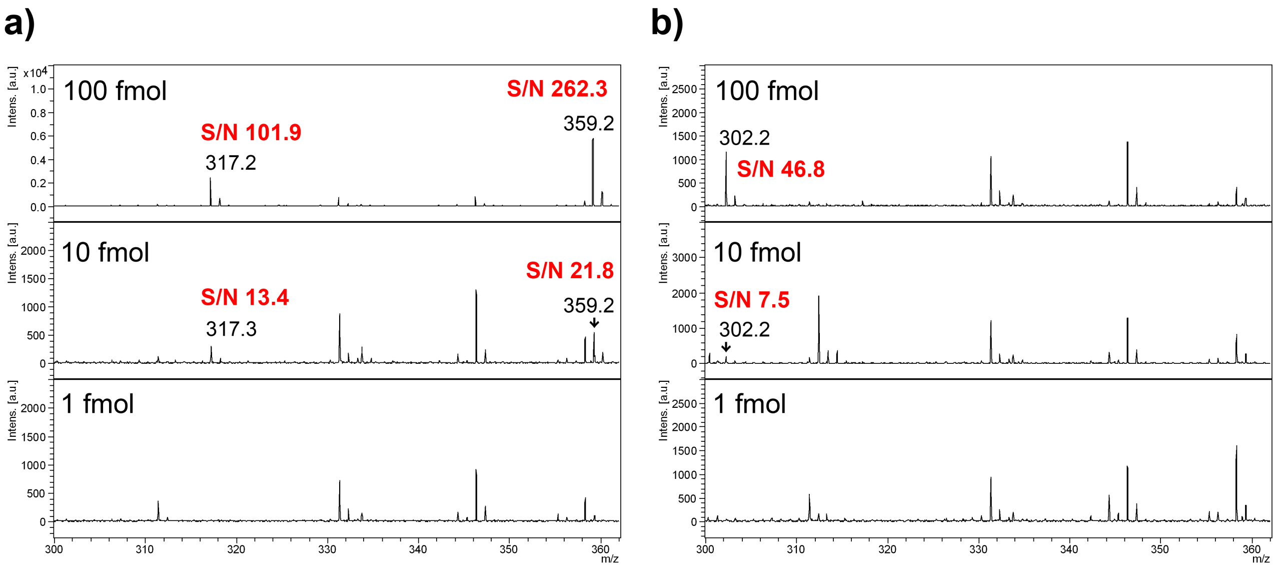

Figure S2. LDI mass spectra of (a) amidopyrene **3** and (b) methyl 4-(1-pyrenyl)butyrate (**9**) in amounts of 100 to 1 fmol. Signal-to-noise (S/N) ratios of the ion peaks $[M–42]^{+•}$ at *m/z* 317.2 and $[M]^{+•}$ at *m/z* 359.2 in (a) and $[M]^{+•}$ at *m/z* 302.2 for **9** in (b) are shown in red.

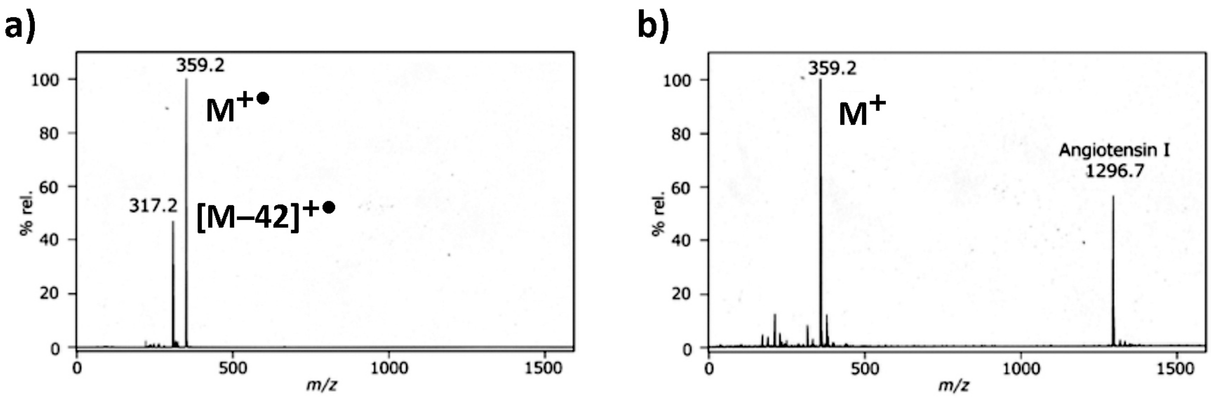

Figure S3. Mass spectra of a mixture of amidopyrene **3** (50 pmol) and the decapeptide angiotensin I [DRVYIHPFHL, *m/z* 1296.7 for $(M+H)^+$] (4 pmol). (a) LDI MS. (b) MALDI-TOF MS using α-CHCA as a matrix. Sample molecular ion peaks were detected with higher sensitivity in (a) than in (b), since no matrix-derived ion peaks were observed.

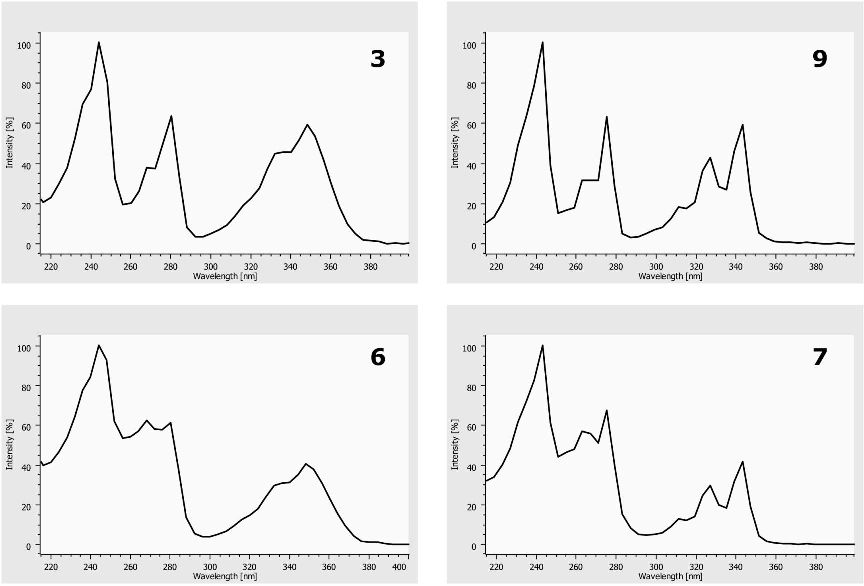

Figure S4. UV absorbance spectra of pyrenes **3** and **9** and the MeOH-adducts of ApA photoaffinity pyrene derivatives **6** and **7**. Data were collected by the analytical HPLC equipped with PDA (photodiode array) monitoring system.

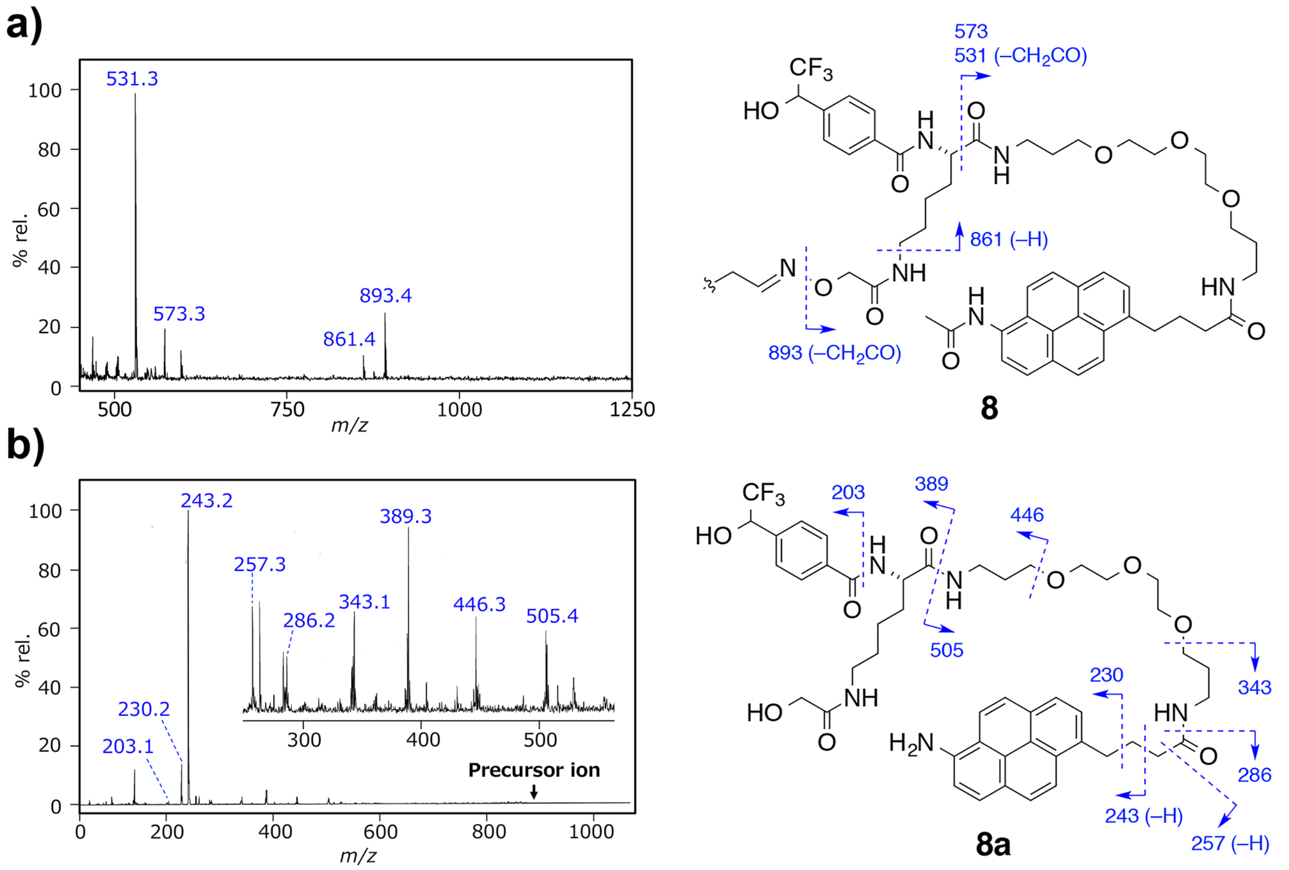

Figure S5. (a) LDI mass spectrum of the water-adduct of ApA–PaP (**8**) (10 pmol). (b) MS/MS analysis of the fragment **8a** generated from **8** (10 pmol). Precursor ion: *m/z* 893.4. Fragmentation mass peaks assigned in (a) and (b) are shown in each chemical structure (right).

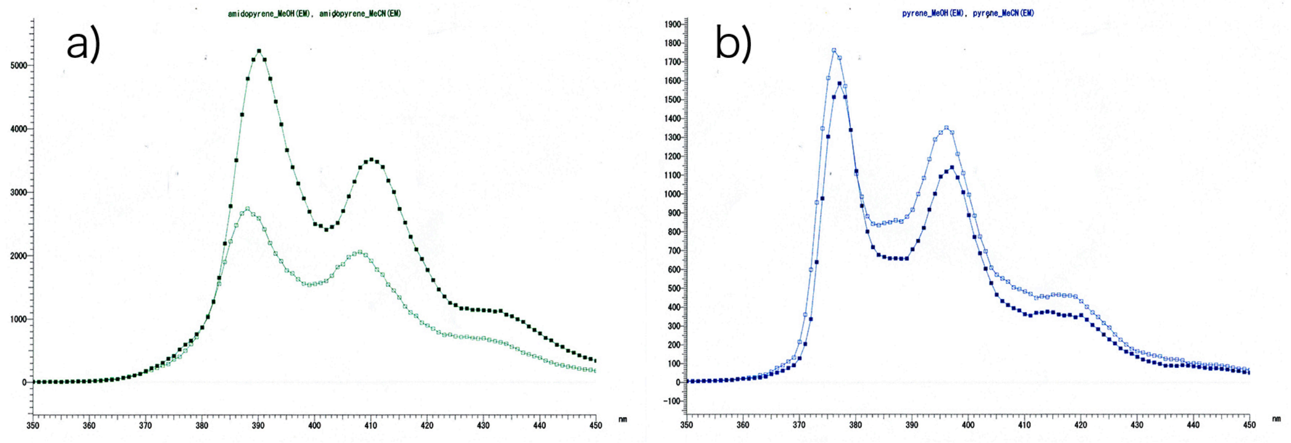

Figure S6. Fluorescence spectra of pyrenes **3** and **9** measured in acetonitrile (solid aquare) and methanol (open square). Excitation wavelength, 337 nm; Concentration, 2.5 μM.

Materials and Methods

General.

NMR spectra were recorded on a Bruker Biospin AVANCE 600 spectrometer (600 MHz for ^{1}H and 150 MHz for ^{13}C) or a Bruker Biospin AVANCE 400 spectrometer (400 MHz for ^{1}H and 100 MHz for ^{13}C). Chemical shifts are reported in parts per million (ppm) with coupling constants (*J*) in hertz relative to the solvent peaks, δ_H 3.31 (residual C$\underline{H}$D$_2$OD), δ_H 7.26 (residual C$\underline{H}$Cl$_3$) and δ_C 77.16 for $\underline{C}$DCl$_3$, δ_H 2.50 (residual C$\underline{H}$D$_2$S(O)CD$_3$) and δ_C 39.52 for ($\underline{C}$D$_3$)$_2$S=O, or δ_H 7.19 (residual C$_5\underline{H}$D$_4$N) and δ_C 150.30 for $\underline{C}_5$HD$_4$N, respectively. For the quantification of minute amounts of specimens by ^{1}H NMR analyses, benzene (10 mM in CD_3OD) was added to the sample solutions as a standard (1:50–1:200, v/v). Optical rotations were measured with a JASCO DIP-1000 polarimeter. IR spectra were recorded on a JASCO FT/IR-230 spectrometer. UV–Vis absorption spectra were measured on a JASCO V-560 spectrometer with MeOH as a solvent. Optical rotations were recorded on a JASCO DIP-1000 polarimeter using the sodium D line. Fluorescence spectra were measured on a Hitachi F-4500 spectrofluorophotometer with acetonitrile or MeOH as solvents. High-resolution electrospray ionization mass spectra (HR-ESIMS) were measured on an AccuTOF CS spectrometer (JEOL). Fuji Silysia silica gels BW-820MH and FL60D were used for column chromatography.

Synthesis and spectroscopic data of the aplyronine A photoaffinity pyrene derivatives.

Methyl 4-(1-pyrenyl)butyrate 9. [S1] To a stirred solution of 1-pyrenebutyric acid (**1**) (196 mg, 0.680 mmol) in dry CH_2Cl_2 (3 mL) were added thionyl chloride (0.39 mL, 5.4 mmol) and MeOH (1.9 mL, 47 mmol) dropwise at –8 °C. After being stirred at room temperature for 2.5 h, the reaction mixture was concentrated *in vacuo*. The crude material was purified with a SiO_2 column (5.8 g, hexane/$CHCl_3$ = 1/1 to 0/1) to give methyl ester **9** (209 mg, quant) as light yellow solid needles. Compound **9**: R_f = 0.55 ($CHCl_3$); mp. 46.8–47.1 °C; ^{1}H NMR (400 MHz, $CDCl_3$) δ 8.31 (d, *J* = 9.3 Hz, 1H), 8.18 (dd, *J* = 7.6, 1.1 Hz, 1H), 8.17 (dd, *J* = 7.6, 1.1 Hz, 1H), 8.12 (d, *J* = 9.3 Hz, 1H), 8.11 (d, *J* = 7.8 Hz, 1H), 8.04 (d, *J* = 9.2 Hz, 1H), 8.03 (d, *J* = 9.2 Hz, 1H), 8.00 (t, *J* = 7.6 Hz, 1H), 7.86 (d, *J* = 7.8 Hz, 1H), 3.71 (s, 3H), 3.40 (t, *J* = 7.7 Hz, 2H), 2.48 (t, *J* = 7.3 Hz, 2H), 2.21 (tt, *J* = 7.7, 7.3 Hz, 2H); ^{13}C NMR (100 MHz, $CDCl_3$) δ 174.1, 135.8, 131.6, 131.0, 130.1, 128.9, 127.6, 127.5, 127.4,
126.8, 126.0, 125.2, 125.1, 125.0, 124.9, 124.9, 123.4, 51.7, 33.8, 32.9, 26.9; IR ($CHCl_3$) 3043, 3011, 2952, 1731, 1604, 1588, 1509, 848 cm^{-1}; HRMS (ESI) *m/z* 325.1209 (calcd for $C_{21}H_{18}NaO_2$ $[M+Na]^+$, Δ +0.5 mmu).

Nitropyrene 10. To a stirred solution of methyl ester **9** (105 mg, 0.347 mmol) in acetic anhydride (2.2 mL) was added 44% aqueous nitric acid (22 μL, 0.20 mmol). The mixture was stirred at room temperature for 12.5 h. After the addition of a second quantity of 44% aqueous nitric acid (22 μL, 0.20 mmol), the mixture was stirred at room temperature for an additional 30 min. After being concentrated *in vacuo*, the crude material was purified with a SiO_2 column (2.2 g, $CHCl_3$) to give a 1:1:1 regioisomer mixture of nitropyrene **10** (128 mg, quant.) as a yellow oil. Compound **10**: R_f = 0.54–0.40 ($CHCl_3$); ^{1}H NMR (400 MHz, $CDCl_3$) δ

8.83 (d, J = 9.4 Hz, 1H), 8.50 (d, J = 9.1 Hz, 1H), 8.36–8.22 (m, 4H), 8.13 (d, J = 9.0 Hz, 1H), 7.97 (d, J = 7.8 Hz, 1H), 3.71 (s, 3H), 3.43 (t, J = 7.7 Hz, 2H), 2.49 (t, J = 7.3 Hz, 2H), 2.20 (tt, J = 7.7, 7.3 Hz, 2H) [6-isomer was only described]; ^{13}C NMR (100 MHz, $CDCl_3$) δ 173.9 (1/3C), 173.8 (1/3C), 173.7 (1/3C), 142.8 (1/3C), 142.6 (1/3C), 142.5 (1/3C), 139.0 (1/3C), 138.6 (1/3C), 135.5 (1/3C), 135.4 (1/3C), 134.8 (1/3C), 133.0 (1/3C), 131.7 (1/3C), 131.0 (1/3C), 130.8 (2/3C), 130.7 (1/3C), 130.4 (1/3C), 129.7 (1/3C), 129.0 (1/3C), 128.6 (1/3C), 128.6 (1/3C), 128.5 (1/3C), 127.9 (1/3C), 127.7 (1/3C), 127.6 (1/3C), 127.5 (1/3C), 127.2 (1/3C), 127.1 (1/3C), 127.1 (1/3C), 126.9 (1/3C), 126.7 (1/3C), 126.2 (1/3C), 125.5 (1/3C), 125.2 (1/3C), 125.2 (1/3C), 124.7 (1/3C), 124.2 (2/3C), 124.1 (1/3C), 124.0 (1/3C), 123.9 (1/3C), 123.5 (1/3C), 123.4 (2/3C), 122.9 (1/3C), 122.8 (1/3C), 122.7 (1/3C), 121.7 (1/3C), 121.6 (1/3C), 121.0 (1/3C), 51.8, (1/3C), 51.8 (2/3C), 33.7 (2/3C), 33.6 (1/3C), 33.1 (1/3C), 32.8 (1/3C), 32.8 (1/3C), 27.1 (2/3C), 26.3 (1/3C); IR ($CHCl_3$) 3027, 2953, 1732, 1587, 1550, 1542, 1509, 1337, 849, 727 cm^{-1}; HRMS (ESI) m/z 370.1029 (calcd for $C_{21}H_{17}NNaO_4$ [M+Na]$^+$, Δ –2.1 mmu).

6-Aminopyrene 2. To the stirred solution of nitropyrene **10** (270 mg, 0.777 mmol) in EtOAc (7 mL) were added acetic acid (0.54 mL) and palladium 10% on carbon (95.6 mg). The mixture was stirred under a hydrogen atmosphere at room temperature for 2.5 h. After the reaction mixture was filtered through a pad of Celite, the residue was washed with EtOAc. The filtrate and the washings were combined and azeotropically concentrated with toluene *in vacuo*. The crude material was purified with a flash SiO_2 column (FL60D 13 g, toluene / EtOAc = 100/1 to 9/1) to give 6-aminopyrene **2** (78.0 mg, 32%) as a yellow oil and a mixture of two other regioisomers (147 mg, 60 %) as an orange oil. Compound **2**: R_f = 0.57 ($CHCl_3$/acetone = 9/1); ^{1}H NMR (400 MHz, $CDCl_3$) δ 8.05–7.99 (br s, 2H), 8.02 (d, J = 9.2 Hz, 1H), 7.97 (d, J = 7.5 Hz, 1H), 7.96 (d, J = 8.1 Hz, 1H), 7.95 (d, J = 9.2 Hz, 1H), 7.93 (d, J = 9.2 Hz, 1H), 7.90 (d, J = 9.2 Hz, 1H), 7.78 (d, J = 7.5 Hz, 1H), 7.37 (d, J = 8.1 Hz, 1H), 3.69 (s, 3H), 3.33 (t, J = 7.7 Hz, 2H), 2.46 (t, J = 7.3 Hz, 2H), 2.19 (tt, J = 7.7, 7.3 Hz, 2H); ^{13}C NMR (100 MHz, $CDCl_3$) δ 174.2, 140.9, 134.7, 130.6, 129.8, 128.5, 127.8, 127.6, 126.4, 126.3, 126.0, 124.3, 123.5, 119.8, 119.5, 117.2, 114.2, 51.7, 33.8, 33.0, 26.6; IR ($CHCl_3$) 3404, 3016, 2976, 2895, 1733, 1624, 1507, 1498, 877, 843, 789 cm^{-1}; UV (MeOH) λ_{max} 362 (ε 21000), 284 (ε 26000), 244 (ε 40000) nm; HRMS (ESI) m/z 340.1322 (calcd for $C_{21}H_{19}NNaO_2$ [M+Na]$^+$, Δ +1.4 mmu).

Amidopyrene 3. To the solution of 6-aminopyrene **2** (371 mg, 1.17 mmol) in dry CH_2Cl_2 (10 mL) was added acetic anhydride (0.13 mL, 1.4 mmol). After being stirred at room temperature for 10 h, the reaction mixture was neutralized with sat. $NaHCO_3$ aq. The organic layer was washed with sat. $NaHCO_3$ aq. (10 mL × 2) and brine, dried with Na_2SO_4, and concentrated *in vacuo*. The crude material was purified with a SiO_2 column (13 g, $CHCl_3$/acetone = 1/0 to 5/1) to give amidopyrene **3** (440 mg, quant.) as a colorless amorphous solid. Compound **3**: R_f = 0.32 ($CHCl_3$/acetone = 9/1); mp. 196.5–209.0 °C; ^{1}H NMR (600 MHz, pyridine-d_5) δ 11.22 (s, 1H), 8.72 (d, J = 8.2 Hz, 1H), 8.60 (d, J = 9.1 Hz, 1H), 8.36 (d, J = 9.2 Hz, 1H), 8.26 (d, J = 8.2 Hz, 1H), 8.14 (d, J = 9.2 Hz, 1H), 8.11 (d, J = 9.1 Hz, 1H), 8.11 (d, J = 7.7 Hz, 1H), 7.86 (d, J = 7.7 Hz, 1H), 3.64 (s, 3H), 3.33 (t, J = 7.5 Hz, 2H), 2.51 (s, 3H), 2.48 (t, J = 7.5 Hz, 2H), 2.18 (tt, J = 7.5, 7.5 Hz, 2H); ^{13}C NMR (150 MHz, pyridine-d_5) δ 174.2, 170.1, 137.1, 136.3, 133.6, 130.7, 129.9, 129.6, 128.5, 128.2 (2C), 126.6, 126.2, 125.8, 125.6, 124.7, 123.6, 122.5, 51.9, 34.2, 33.4, 27.7, 24.6; IR ($CHCl_3$) 3427, 3030, 3009, 2953, 1731, 1685, 1550, 1519, 1484, 847, 720 cm^{-1}; UV (MeOH) λ_{max} 346 (ε 26000), 279 (ε 29000), 243 (ε 38000) nm; HRMS (ESI) m/z 382.1439 (calcd for $C_{23}H_{21}NNaO_3$ [M+Na]$^+$, Δ +2.5 mmu).

Carboxylic acid 11. To the solution of amidopyrene **3** (65.7 mg, 0.183 mmol) in dry THF (1 mL) was added 1 M LiOH aq. (2 mL). After being stirred at room temperature for 3.5 h, the reaction mixture was acidified with 1 M HCl aq. and extracted with EtOAc (10 mL × 5). The combined extracts were washed with brine, dried with Na_2SO_4, and concentrated *in vacuo*. The crude material was absorbed on SiO_2 (0.4 g) and purified with a SiO_2 column (2.4 g, $CHCl_3$/acetone = 2/1, 1/1, 1/2 to 0/1) to give carboxylic acid **11** (51 mg, 81%) as a white powder. Compound **11**: R_f = 0.49 ($CHCl_3$/acetone = 1/1); mp. 252.8–254.0 °C; ^{1}H NMR (600 MHz, DMSO-d_6) δ 12.10 (br s, 1H), 10.29 (s, 1H), 8.34 (d, J = 9.1 Hz, 1H), 8.27–8.22 (m, 3H), 8.21 (d, J = 7.8 Hz, 1H), 8.17 (d, J = 9.3 Hz, 1H), 8.16 (d, J = 9.3 Hz, 1H), 7.93 (d, J = 7.8 Hz, 1H), 3.34 (t, J = 7.4 Hz, 2H), 2.38 (t, J = 7.2 Hz, 2H), 2.27 (s, 3H), 2.00 (tt, J = 7.4, 7.2 Hz, 2H); ^{13}C NMR (150 MHz, DMSO-d_6) δ 174.4, 169.1, 136.4, 131.8, 129.2, 128.4, 128.0, 127.7, 127.2, 127.1, 124.8, 124.7, 124.6, 124.4, 124.0, 123.4, 122.6, 121.5, 33.4, 32.1, 26.8, 23.6; IR (KBr) 3282, 3042, 2952, 1698, 1654, 1604, 1552, 1521, 1499, 844, 715, 682 cm^{-1}; HRMS (ESI) *m/z* 344.1297 (calcd for $C_{22}H_{18}NO_3$ [M–H]$^-$, Δ +0.5 mmu).

Amide 13. To a solution of carboxylic acid **11** (14.2 mg, 41.1 μmol) in dry CH_2Cl_2 (1 mL) were added HOBt (13.1 mg, 97.0 μmol), EDC·HCl (14.1 mg, 73.6 μmol), and a solution of amine **12** [S2] (17.9 mg, 55.9 μmol) in dry CH_2Cl_2 (1 mL). After being stirred for 21 h, the reaction mixture was washed with sat. NH_4Cl aq. (10 mL × 3) and brine, dried with Na_2SO_4, and concentrated *in vacuo*. The crude material was purified with a SiO_2 column (1.0 g, $CHCl_3$/acetone = 9/1, 5/1 to 1/1) to give amide **13** (19.3 mg, 72%) as a yellow amorphous solid. Compound **13**: R_f = 0.36 ($CHCl_3$/acetone = 9/1); mp. 131.9–140.5 °C; ^{1}H NMR (600 MHz, CD_3OD) δ 8.34 (d, J = 9.3 Hz, 1H), 8.20 (d, J = 8.2 Hz, 1H), 8.16 (d, J = 9.1 Hz, 1H), 8.16 (d, J = 7.8 Hz, 1H), 8.14 (d, J = 9.3 Hz, 1H), 8.12 (d, J = 9.1 Hz, 1H), 8.11 (d, J = 8.2 Hz, 1H), 7.93 (d, J = 7.8 Hz, 1H), 3.64 (m, 1H), 3.58 (m, 1H), 3.54 (m, 4H), 3.51 (m, 2H), 3.50 (t, J = 6.1 Hz, 2H), 3.44 (m, 2H), 3.40 (t, J = 6.1 Hz, 2H), 3.28 (t, J = 6.7 Hz, 2H), 3.06 (t, J = 6.8 Hz, 2H), 2.37 (s, 3H), 2.35 (t, J = 7.3 Hz, 2H), 2.17 (tt, J = 7.5, 7.2 Hz, 2H), 1.76 (tt, J = 6.4, 6.4 Hz, 2H), 1.64 (tt, J = 7.3, 7.5 Hz, 2H), 1.40 (s, 9H); ^{13}C NMR (100 MHz, $CDCl_3$) δ 172.7, 169.4, 156.2, 136.7, 130.4, 129.7, 129.2, 129.0, 128.1, 127.8, 127.3, 125.6, 125.3, 125.0 (2C), 124.4, 123.1, 123.0, 120.0, 79.2, 77.4, 70.5, 70.4, 70.4, 70.2 (2C), 70.1, 69.4, 36.2, 33.0, 29.7, 29.0, 28.6 (3C), 27.6, 24.4; IR ($CHCl_3$) 3450, 3009, 2982, 2931, 2872, 1694, 1661, 1655, 1517, 1499, 846, 788 cm^{-1}; HRMS (ESI) *m/z* 670.3485 (calcd for $C_{37}H_{49}N_3NaO_7$ [M+Na]$^+$, Δ +2.2 mmu).

Amide 14. Prepared from 1-pyrenebutylic acid (**1**) in 84% yield similarly as described for **13**. Compound **14**: R_f = 0.46 ($CHCl_3$/acetone = 2/1); ^{1}H NMR (600 MHz, $CDCl_3$) δ 8.30 (d, J = 9.3 Hz, 1H), 8.16 (dd, J = 1.3, 7.5 Hz, 1H), 8.15 (dd, J = 1.3, 7.5 Hz, 1H), 8.10 (d, J = 9.3 Hz, 1H), 8.10 (d, J = 7.7 Hz, 1H), 8.02 (d, J = 9.0 Hz, 1H), 8.01 (d, J = 9.0 Hz, 1H), 7.98 (t, J = 7.5 Hz, 1H), 7.86 (d, J = 7.7 Hz, 1H), 6.24 (br s, 1H), 4.88 (br s, 1H), 3.56–3.48 (m, 6H), 3.44–3.34 (m, 10H), 3.15 (m, 2H), 2.27 (t, J = 7.2 Hz, 2H), 2.20 (tt, J = 7.2, 7.2 Hz, 2H), 1.76 (tt, J = 6.0, 6.0 Hz, 2H), 1.66 (tt, J = 6.2, 6.2 Hz, 2H), 1.48 (s, 9H); ^{13}C NMR (150 MHz, $CDCl_3$) δ 172.7, 156.1, 136.2, 131.5, 131.0, 130.0, 128.9, 127.6, 127.5, 127.4, 126.8, 126.0, 125.2, 125.1, 125.0, 124.9, 124.9, 123.6, 79.1, 70.5, 70.5, 70.3, 70.2, 70.2, 69.5, 38.6, 38.2, 36.2, 33.0, 29.7, 29.1, 28.6 (3C), 27.6; IR ($CHCl_3$) 3452, 3399, 3007, 2950, 2931, 2873, 1705, 1658, 1510, 1455, 848, 766 cm^{-1}; HRMS (ESI) *m/z* 613.3241 (calcd for $C_{35}H_{46}N_2NaO_6$ [M+Na]$^+$, Δ –0.7mmu).

Lysine amide 16. A solution of amide **13** (5.7 mg, 8.8 μmol) in a 1:1 mixture of dry CH_2Cl_2 and trifluoroacetic acid (1 mL) was stirred for 30 min at room temperature and azeotropically concentrated with toluene *in vacuo* to give an amine TFA salt. Triethylamine (4 μL, 29 μmol) was added to a stirred solution of

phthalimide (Phth)-protected carboxylic acid **15** (7.1 mg, 16 μmol),[S3] HOBt (3.1 mg, 23 μmol), EDC·HCl (2.8 mg, 15 μmol), and the above amine TFA salt in dry CH_2Cl_2 (3 mL). After being stirred for 50 h, the reaction mixture was washed with sat. $NaHCO_3$ aq. and brine, dried with Na_2SO_4, and concentrated *in vacuo*. The crude material was purified with a SiO_2 column (0.6 g, $CHCl_3$/acetone = 2/1 to 1/1) to give lysine amide **16** (6.5 mg, 76%) as a yellow oil. Compound **16**: R_f = 0.20 ($CHCl_3$/acetone = 1/1); $[\alpha]_D^{18}$ –0.42 (*c* 0.26, $CHCl_3$); ^{1}H NMR (600 MHz, DMSO-d_6) δ 10.29 (br s, 1 H), 8.31 (d, *J* = 9.1 Hz, 1H), 8.31 (s, 1H), 8.26–8.21 (m, 2H), 8.20 (d, *J* = 7.8 Hz, 1H), 8.17 (d, *J* = 8.9 Hz, 1H), 8.15 (d, *J* = 9.1 Hz, 1H), 8.14 (m, 1H), 8.09 (t, *J* = 5.6 Hz, 1H), 8.03 (t, *J* = 5.5 Hz, 1H), 7.92 (d, *J* = 7.8 Hz, 1H), 7.89–7.84 (m, 2H), 7.83–7.80 (m, 2H), 7.75 (t, *J* = 5.8 Hz, 1H), 4.59 [4.56, 4.53] (s, 2H), 3.80 (m, 1H), 3.50–3.27 (m, 14H), 3.13–2.99 (m, 6H), 2.27 [2.30] (m, 3H), 2.22 [2.21] (t, *J* = 7.3 Hz, 2H), 2.00 (tt, *J* = 7.3, 7.7 Hz, 2H), 1.62 (m, 2H), 1.58 (m, 2H), 1.50–1.37 (m, 2H), 1.32–1.09 (m, 4H), 1.35 (s, 9H); ^{13}C NMR (150 MHz, DMSO-d_6) δ 171.8, 169.1, 168.0, 167.7, 165.7, 162.9, 162.9, 136.6, 134.9 (2C), 134.4, 131.8, 131.6, 129.2, 128.9, 128.5, 128.4, 128.2, 127.7, 127.1 (2C), 124.7, 124.6, 124.4, 123.4, 123.4, 123.1, 122.6, 121.5, 79.2, 75.8, 69.7, 69.7, 69.5, 69.4, 68.5, 68.1, 55.8, 38.2, 35.8, 35.0, 32.4, 32.1, 29.6 (3C), 29.4, 28.6, 28.2, 27.5, 23.5, 22.9, 21.0; IR ($CHCl_3$) 3425, 3378, 3006, 2929, 2868, 1734, 1716, 1670, 1539, 1522, 1370, 846, 703 cm^{-1}; HRMS (ESI) *m/z* 1001.4618 (calcd for $C_{53}H_{66}N_6NaO_{12}$ $[M+Na]^+$, Δ –1.3 mmu).

Lysine amide 17. Prepared from amide **14** in 81% yield similarly as described for **16**. Compound **17**: R_f = 0.20 ($CHCl_3$/acetone = 2/1); $[\alpha]_D^{29}$ –0.13 (*c* 0.59, $CHCl_3$); ^{1}H NMR (400 MHz, $CDCl_3$) δ 8.29 (d, *J* = 9.3 Hz, 1H), 8.15 (d, *J* = 7.6 Hz, 1H), 8.14 (d, *J* = 7.6 Hz, 1H), 8.09 (d, *J* = 9.3 Hz, 1H), 8.09 (d, *J* = 7.8 Hz, 1H), 8.01 (d, *J* = 9.2 Hz, 1H), 7.99 (d, *J* = 9.2 Hz, 1H), 7.97 (t, *J* = 7.6 Hz, 1H), 7.86 (d, *J* = 7.8 Hz, 1H), 7.79 (dd, *J* = 3.1, 5.5 Hz, 2H), 7.70 (dd, *J* = 3.1, 5.5 Hz, 2H), 6.74 (br s, 1H), 6.37 (br s, 1H), 5.29 (br d, *J* = 7.4 Hz, 1H), 4.65 (s, 2H), 4.01 (br s, 1H), 3.55–3.51 (m, 6H), 3.50–3.20 (m, 15H), 2.29 (t, *J* = 7.2 Hz, 2H), 2.20 (tt, *J* = 7.2, 7.2 Hz, 2H), 1.82 (m, 2H), 1.77 (tt, *J* = 6.2, 6.2 Hz, 2H), 1.70 (tt, *J* = 6.1, 6.1 Hz, 2H), 1.58 (m, 2H), 1.41 (s, 9H), 1.39 (m, 2H); ^{13}C NMR (100 MHz, $CDCl_3$) δ 172.9, 172.1, 167.1, 163.8 (2C), 155.8, 136.2, 135.1 (2C), 131.5, 131.0, 130.0, 128.9, 128.5 (2C), 127.6, 127.5, 127.5, 126.8, 126.0, 125.2, 125.1, 125.0, 124.9, 124.9, 124.1 (2C), 123.6, 79.9, 77.4, 76.9, 70.5, 70.2, 70.0, 69.8, 69.7, 54.6, 38.9, 37.9, 36.2, 33.0, 32.4, 29.4, 29.2, 29.1, 29.0, 28.5 (3C), 27.7, 22.9; IR ($CHCl_3$) 3432, 3376, 3009, 2977, 2931, 2893, 1734, 1716, 1706, 1669, 1657, 1540, 1522, 1370, 848, 707 cm^{-1}; HRMS (ESI) *m/z* 944.4423 (calcd for $C_{51}H_{63}N_5NaO_{11}$ $[M+Na]^+$, Δ +0.7 mmu).

Diazirine amide 19. A solution of lysine amide **16** (11.4 mg, 11.6 μmol) in a 1:1 mixture of dry CH_2Cl_2 and trifluoroacetic acid (1 mL) was stirred for 1 h at room temperature and azeotropically concentrated with toluene *in vacuo* to give an amine TFA salt. Diazirine succinyl ester **18** [S4] (9.3 mg, 28.4 μmol) in dry DMF (1 mL) and triethylamine (9.0 μL, 16 μmol) were added to a stirred solution of the above amine TFA salt in dry DMF (0.5 mL). After being stirred for 17.5 h at room temperature, the resulting mixture was concentrated *in vacuo*. The crude material was purified by a reversed-phase HPLC [Develosil ODS-HG-5 (ϕ 20 mm I.D. × 250 mm), 5 mL/min, UV 254 nm, 55% aq. MeCN, t_R = 29–32 min] to give diazirine amide **19** (10.2 mg, 88%) as a yellow oil. Compound **19**: $[\alpha]_D^{18}$ +1.5 (*c* 0.69, $CHCl_3$); ^{1}H NMR (600 MHz, DMSO-d_6) δ 10.29 (s, 1H), 8.55 (d, *J* = 7.8 Hz, 1H), 8.30 (d, *J* = 9.2 Hz, 1H), 8.25–8.21 (m, 3H), 8.19 (d, *J* = 7.8 Hz, 1H), 8.16 (d, *J* = 8.2 Hz, 1H), 8.15 (d, *J* = 9.2 Hz, 1H), 7.99 (d, *J* = 8.2 Hz, 2H), 7.95 (t, *J* = 5.7 Hz, 1H), 7.92 (d, *J* = 7.7 Hz, 1H), 7.85 (s, 4H), 7.82 (t, *J* = 5.6 Hz, 1H), 7.34 (d, *J* = 8.2 Hz, 2H), 4.58 (s, 2H), 4.35 (dt,

J = 5.4, 8.5 Hz, 1H), 3.85–3.42 (m, 10H), 3.40 (m, 1H), 3.38 (t, J = 5.8 Hz, 2H), 3.35 (t, J = 6.4 Hz, 2H), 3.28 (t, J = 7.6 Hz, 2H), 3.17–3.03 (m, 4H), 2.26 (s, 3H), 2.21 (t, J = 7.3 Hz, 2H), 1.99 (tt, J = 7.6, 7.3 Hz, 2H), 1.72 (m, 2H), 1.62 (tt, J = 6.7, 6.7 Hz, 2H), 1.60 (tt, J = 6.7, 6.2 Hz, 2H), 1,46 (m, 2H), 1.37 (m, 1H), 1.29 (m, 1H); ^{13}C NMR (150 MHz, DMSO-d_6) δ 171.8, 171.5, 169.1, 165.7, 165.3, 162.9 (2C), 136.6, 135.8, 134.9 (2C), 131.8, 130.2, 129.2, 128.5, 128.5 (2C), 128.4 (2C), 128.3, 128.0, 127.7, 127.1 (2C), 126.2 (2C), 124.7, 124.6, 124.4, 124.0, 123.4 (2C), 123.4, 122.7, 121.7 (q, $^1J_{CF}$ = 273 Hz), 121.5, 75.8, 69.7, 69.7, 69.5, 69.5, 68.1, 68.0, 53.6, 38.2, 35.9, 35.8, 35.0, 32.3, 31.2, 29.4, 29.2, 28.5, 28.0 (q, $^2J_{CF}$ = 40 Hz), 27.5, 23.5, 23.1; IR ($CHCl_3$) 3421, 3370, 3006, 2944, 2870, 1735, 1669, 1655, 1557, 1541, 1523, 1372, 1344, 846, 705 cm^{-1}; HRMS (ESI) *m/z* 1113.4309 (calcd for $C_{57}H_{61}F_3N_8NaO_{11}$ $[M+Na]^+$, Δ +0.5 mmu).

Diazirine amide 20. Prepared from lysine amide **17** in 51% yield similarly as described for **19**. Compound **20**: $[\alpha]_D^{30}$ +0.16 (c 0.38, $CHCl_3$); ^{1}H NMR (600 MHz, $CDCl_3$) δ 8.26 (d, J = 9.2 Hz, 1H), 8.14 (d, J = 7.6 Hz, 1H), 8.14 (d, J = 7.6 Hz, 1H), 8.08 (d, J = 9.2 Hz, 1H), 8.08 (d, J = 7.7 Hz, 1H), 8.01 (d, J = 9.2 Hz, 1H), 8.00 (d, J = 9.2 Hz, 1H), 7.97 (dd, J = 7.6, 7.6 Hz, 1H), 7.83 (d, J = 7.7 Hz, 1H), 7.80 (d, J = 8.4 Hz, 2H), 7.74 (dd, J = 3.1, 5.5 Hz, 2H), 7.69 (dd, J = 3.1, 5.5 Hz, 2H), 7.22 (d, J = 7.6 Hz, 1H), 7.15 (d, J = 8.4 Hz, 2H), 6.97 (br t, J = 5.5 Hz, 1H), 6.44 (br t, J = 5.3 Hz, 1H), 4.65 (d, J = 16.2 Hz, 1H), 4.60 (d, J = 16.2 Hz, 1H), 3.56–3.52 (m, 6H), 3.47–3.26 (m, 15H), 2.27 (t, J = 6.8 Hz, 2H), 2.18 (tt, J = 7.4, 7.4 Hz, 2H), 1.95 (m, 2H), 1.82–1.75 (m, 3H), 1.71 (tt, J = 6.0, 6.0 Hz, 2H), 1,60 (m, 2H), 1.44 (m, 2H); ^{13}C NMR (150 MHz, $CDCl_3$) δ 172.9, 171.3, 167.1, 166.0, 163.7 (2C), 136.2, 135.2, 135.1 (3C), 132.5, 131.5, 131.0, 130.0, 128.9, 128.5 (2C), 127.8, 127.6, 127.5, 127.5, 126.8, 126.6 (2C), 126.0, 125.2, 125.1, 125.0, 124.9, 124.9, 124.1 (2C), 123.6, 122.0 (q, $^1J_{CF}$ = 273 Hz), 70.5, 70.4, 70.2, 70.1, 70.0, 70.0, 69.9, 53.7, 38.8, 38.2, 37.9, 36.2, 33.0, 32.4, 29.2, 29.1, 28.9, 28.4 (q, $^2J_{CF}$ = 41 Hz), 27.7, 22.6; IR ($CHCl_3$) 3420, 3371, 3004, 2949, 2871, 1734, 1717, 1670, 1654, 1558, 1541, 1523, 1374, 1340, 848, 706 cm^{-1}; HRMS (ESI) *m/z* 1056.4085 (calcd for $C_{55}H_{58}F_3N_7NaO_{10}$ $[M+Na]^+$, Δ –0.4 mmu).

Alkoxyamine 21. Diazirine amide **19** (1.0 mg, 0.92 μmol) was dissolved in a 21 mM solution of hydrazine monohydrate in EtOH (1.0 mL, 21 μmol). After being stirred at room temperature for 50 min, the resulting mixture was azeotropically concentrated with toluene *in vacuo* to give alkoxyamine **21** (quant. monitored by TLC analysis: R_f = 0.20, $CHCl_3$/acetone = 1/4), which was immediately used for the next step without further purification.

Alkoxyamine 22. Prepared from diazirine amide **20** similarly as described for **21**, and was immediately used for the next step without further purification.

Photoaffinity amidopyrene derivative ApA-PaP (4). Aplyronine A (ApA) was isolated from the sea hare *Aplysia kurodai*, as described previously [27]. A solution of ApA (0.20 mg, 189 nmol) in a 3:1 mixture of 1,4-dioxane (100 μL) and 2 M aq. HCl (33 μL) was stirred for 85 min at 50 °C. The resulting mixture was diluted with sat. $NaHCO_3$ aq. (150 μL) and water (1 mL), and extracted with $CHCl_3$ (1 mL × 5). The combined extracts were washed with brine and concentrated *in vacuo* to give an aldehyde, which was used for the next step without further purification.

A solution of the aldehyde and the alkoxyamine **21** prepared as above in a 3:2 mixture of EtOH and 50 mM acetate buffer (pH 4.0) (0.5 mL) was stirred at room temperature for 27 h. The reaction mixture was directly applied to a Develosil ODS-HG-5 HPLC column (ϕ 20 mm I.D. × 250 mm). Samples were eluted with MeOH / 20 mM ammonium acetate (85:15) at a flow rate of 5 mL/min and with monitoring at 254 nm

to give ApA–PaP (**4**) (92 nmol, 48%, based on NMR quantification, t_R = 32–35 min, *E*/*Z* = 7/3 for the C34 isomers). Compound **4**: ^{1}H NMR (600 MHz, CD_3OD) δ 8.34 (d, *J* = 9.2 Hz, 1H), 8.20 (d, *J* = 8.2 Hz, 1H), 8.17 (d, *J* = 9.2 Hz, 1H), 8.16 (d, *J* = 7.9 Hz, 1H), 8.14 (d, *J* = 9.2 Hz, 1H), 8.12 (d, *J* = 7.8 Hz, 1H), 8.12 (d, *J* = 9.4 Hz, 1H), 7.94 (d, *J* = 8.3 Hz, 2H), 7.92 (d, *J* = 7.9 Hz, 1H), 7.52 [6.79]^1 (dd, *J* = 7.0, 5.7 Hz, 1H), 7.31 (d, *J* = 8.3 Hz, 2H), 7.19 (br dd, *J* = 15.1, 10.9 Hz, 1H), 6.35 (m, 1H), 6.20 (m, 1H), 5.96 (d, *J* = 15.1 Hz, 1H), 5.61 (ddd, *J* = 14.8, 10.4, 3.8 Hz, 1H), 5.54 (br d, *J* = 11.3 Hz, 1H), 5.09 (m, 1H), 4.97 (m, 1H), 4.82 (m, 1H), 4.78 (dd, *J* = 10.1, 2.5 Hz, 1H), 4.67 (m, 1H), 4.43 (m, 1H), 4.38 [4.43]^1 (s, 2H), 3.70–3.63 (m, 2H), 3.54–3.48 (m, 5H), 3.50 (t, *J* = 6.1 Hz, 2H), 3.48 (m, 2H), 3.42 (m, 2H), 3.41 (t, *J* = 6.4 Hz, 2H), 3.38–3.33 (m, 2H), 3.37 [3.37]^1 (s, 3H), 3.32 (m, 2H), 3.27 (t, *J* = 6.7 Hz, 2H), 3.25–3.19 (m, 5H), 3.18 (s, 3H), 3.14 [3.13]^2 (s, 3H), 3.08 (dd, *J* = 9.4, 2.5 Hz,1H), 2.54–2.41 (m, 2H), 2.38 [2.38]^2 (s, 3H), 2.38 [2.37]^3 (s, 3H), 2.34 [2.34]^3 (s, 3H), 2.32 [2.33]^2 (s, 3H), 2.33–2.20 (m, 2H), 2.19–2.10 (m, 2H), 2.16 (t, *J* = 7.7 Hz, 2H), 2.08–1.95 (m, 2H), 2.03 [2.02]^2 (s, 3H), 2.01 (s, 3H), 1.93–1.81 (m, 2H), 1.79–1.65 (m, 5H), 1.75 (tt, *J* = 7.7, 7.3 Hz, 2H), 1.69 (tt, *J* = 6.4, 6.4 Hz, 2H), 1.64–1.57 (m, 4H), 1.56–1.47 (m, 10H), 1.43–1.21 (m, 4H), 1.32 (d, *J* = 6.9 Hz, 3H), 1.29 (d, *J* = 7.0 Hz, 3H), 1.28 (d, *J* = 7.1 Hz, 3H), 1.17–1.08 (m, 2H), 1.02 (d, *J* = 6.6 Hz, 3H), 0.99 (d, *J* = 6.7 Hz, 3H), 0.97 [0.98]^3 (d, *J* = 7.1 Hz, 3H), 0.90 [0.88]^2 (d, *J* = 6.9 Hz, 3H), 0.76 [0.74]^3 (d, *J* = 5.7 Hz, 3H) Chemical shifts of the minor diastereomers are within parentheses as follows: []^1, 7:3 at C34 stereoisomers; []^2, 3:1 at C7 trimethylserine moiety; []^3, 1.4:1 at C29 dimethylalanine moiety; HRMS (ESI) *m/z* 1011.5508 (calcd for $(C_{106}H_{155}F_3N_{10}Na_2O_{22})/2$ $[M+2Na]^{2+}$, Δ –1.9 mmu).

Photoaffinity pyrene derivative ApA-PP (5). Prepared from ApA and diazirine amide **22** in 66% yield similarly as described for **4**. Compound **5**: ^{1}H NMR (600 MHz, CD_3OD) δ 8.34 (d, *J* = 9.3 Hz, 1H), 8.19 (d, *J* = 7.1 Hz, 1H), 8.18 (d, *J* = 7.1 Hz, 1H), 8.17 (d, *J* = 9.3 Hz, 1H), 8.14 (d, *J* = 7.7 Hz, 1H), 8.05 (d, *J* = 8.9 Hz, 1H), 8.03 (d, *J* = 8.9 Hz, 1H), 7.99 (dd, *J* = 7.1, 7.1 Hz, 1H), 7.92 (d, *J* = 8.4 Hz, 2H), 7.90 (d, *J* = 7.7 Hz, 1H), 7.51 [6.78]^1 (dd, *J* = 6.4, 6.4 Hz, 1H), 7.30 (d, *J* = 8.4 Hz, 2H), 7.18 (dd, *J* = 11.1, 15.1 Hz, 1H), 6.33 (m, 1H), 6.19 (m, 1H), 5.94 (d, *J* = 15.1 Hz, 1H), 5.59 (m, 1H), 5.53 (br d, *J* = 10.7 Hz, 1H), 5.08 (m, 1H), 4.96 (m, 1H), 4.80 (m, 1H), 4.75 (m, 1H), 4.65 (m, 1H), 4.43 (m, 1H), 4.37 [4.42]^1 (s, 2H), 3.66–3.62 (m, 2H), 3.56–3.46 (m, 7H), 3.49 (t, *J* = 6.2 Hz, 2H), 3.43–3.32 (m, 8H), 3.36 [3.36]^2 (s, 3H), 3.27–3.18 (m, 7H), 3.17 (s, 3H), 3.13 [3.12]^2 (s, 3H), 3.06 (dd, *J* = 2.7, 9.6 Hz, 1H), 2.52–2.28 (m, 4H), 2.36 [2.37]^2 (s, 6H), 2.32 [2.30]^1 [2.33]^3 [2.31]^{1,3} (s, 6H), 2.34–2.30 (m, 2H), 2.26–2.20 (m, 1H), 2.16 (t, *J* = 7.6, *J* = 7.6 Hz, 2H), 2.18–2.08 (m, 1H), 2.05–1.96 (m, 4H), 2.01 [2.03]^1 [2.00]^3 [2.02]^{1,3} (s, 3H), 1.84 (m, 1H), 1.79–1.48 (m, 11H), 1.74 (tt, *J* = 6.4, 6.4 Hz, 2H), 1.70 (tt, *J* = 6.4, 6.4 Hz, 2H), 1.62–1.57 (m, 2H), 1.49 [1.50]^2 (s, 3H), 1.43–1.21 (m, 11H), 1.31 (d, *J* = 6.8 Hz, 3H), 1.17–1.07 (m, 3H), 1.02 (d, *J* = 6.7 Hz, 3H), 0.99 (d, *J* = 6.8 Hz, 3H), 0.96 (d, *J* = 7.0 Hz, 3H), 0.89 [0.86]^2 (d, *J* = 6.9 Hz, 3H), 0.75 [0.73]^3 (d, *J* = 5.7 Hz, 3H). Chemical shifts of the minor diastereomers are within parentheses as follows: []^1, 7:3 at C34 stereoisomers; []^2, 3:1 at C7 trimethylserine moiety; []^3, 1.4:1 at C29 dimethylalanine moiety; HRMS (ESI) *m/z* 983.0446 (calcd for $(C_{104}H_{152}F_3N_9Na_2O_{21})/2$ $[M+2Na]^{2+}$, Δ+2.6 mmu).

MeOH-adduct of ApA-PaP (6). HRMS (ESI) *m/z* 1013.5618 (calcd for $C_{107}H_{159}F_3N_8Na_2O_{23}$ $[M+2Na]^{2+}$, Δ +0.9 mmu).

MeOH-adduct of ApA-PP (7). HRMS (ESI) *m/z* 963.0726 (calcd for $C_{105}H_{158}F_3N_7O_{22}$ $[M+2H]^{2+}$, Δ +2.3 mmu).

Water-adduct of ApA-PaP (8). HRMS (ESI) *m/z* 984.5734 (calcd for $C_{106}H_{159}F_3N_8O_{23}$ $[M+2H]^{2+}$, Δ +0.4 mmu).

Supporting references

S1. Gorteau, V. *et al.* Synthetic multifunctional pores with external and internal active sites for ligand gating and noncompetitive blockage. *J. Am. Chem. Soc.* **126**, 13592–13593 (2004).

S2. Wilbur, S. D., Pathare, P. M., Weerawarna, S. A. & Hamlin, D. K. Biotin-containing compounds, biotinylation reagents and methods. *PCT Int. Appl.* WO9729114.

S3. Clavé, G. *et al.* A novel heterotrifunctional peptide-based cross-linking reagent for facile access to bioconjugates. Applications to peptide fluorescent labelling and immobilization. *Org. Biomol. Chem.* **6**, 3065–3078 (2008).

S4. Strømgaard, K. *et al.* Ginkgolide derivatives for photolabeling studies: preparation and pharmacological evaluation. *J. Med. Chem.* **45**, 4038–4046 (2002).

NMR charts

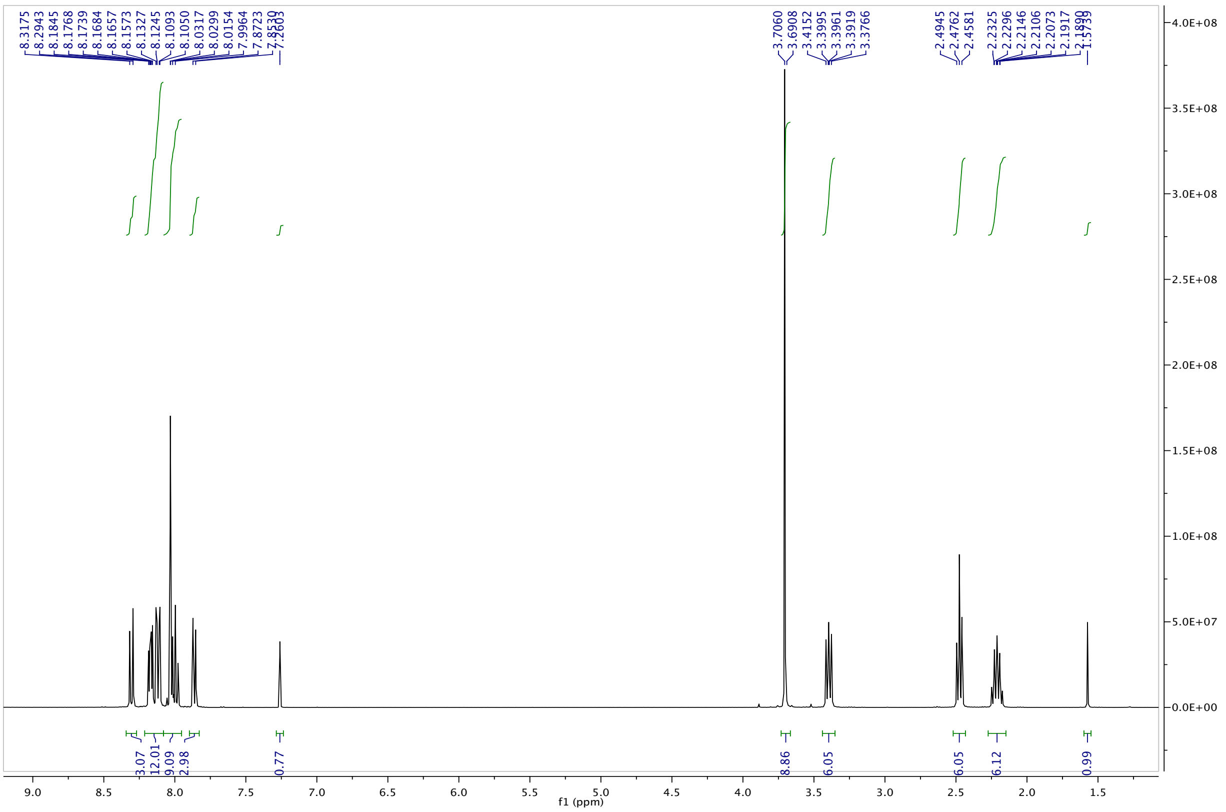

^{1}H NMR spectrum of methyl ester **9** (400 MHz, $CDCl_3$).

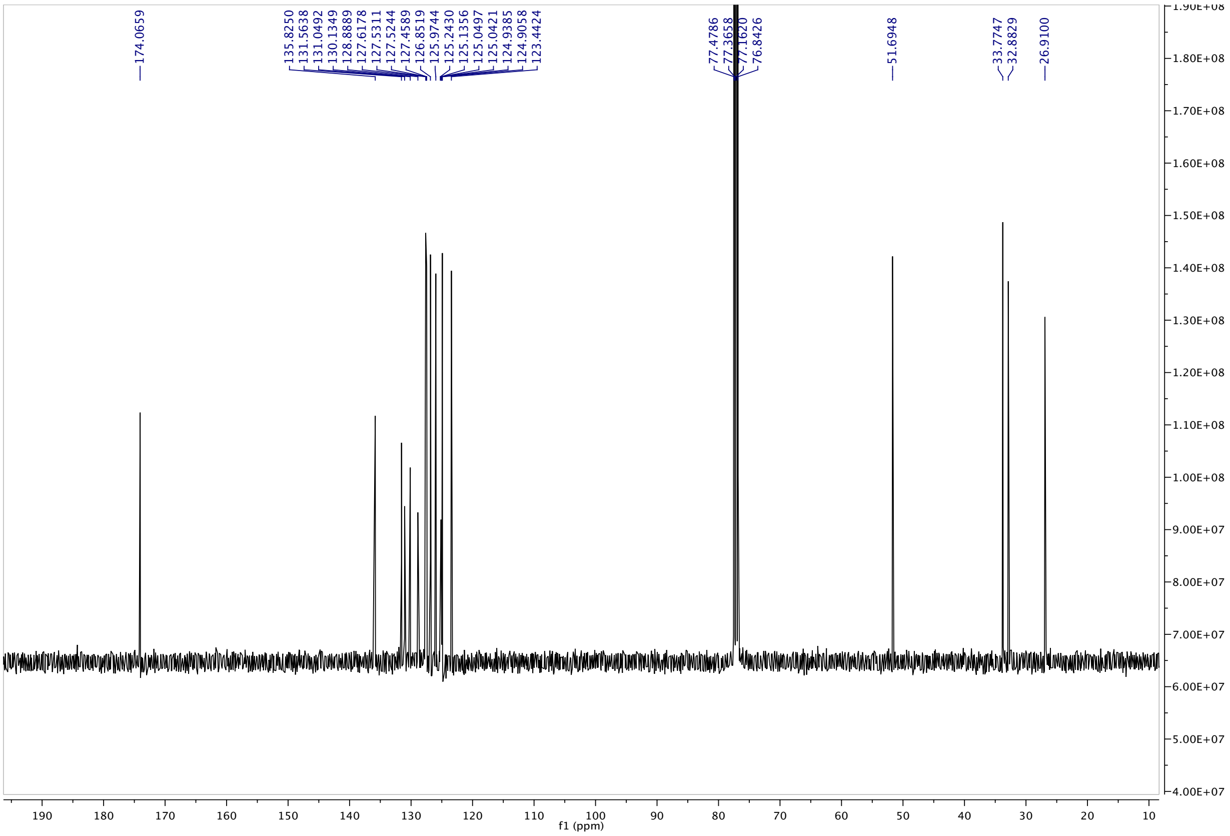

^{13}C NMR spectrum of methyl ester **9** (100 MHz, $CDCl_3$).

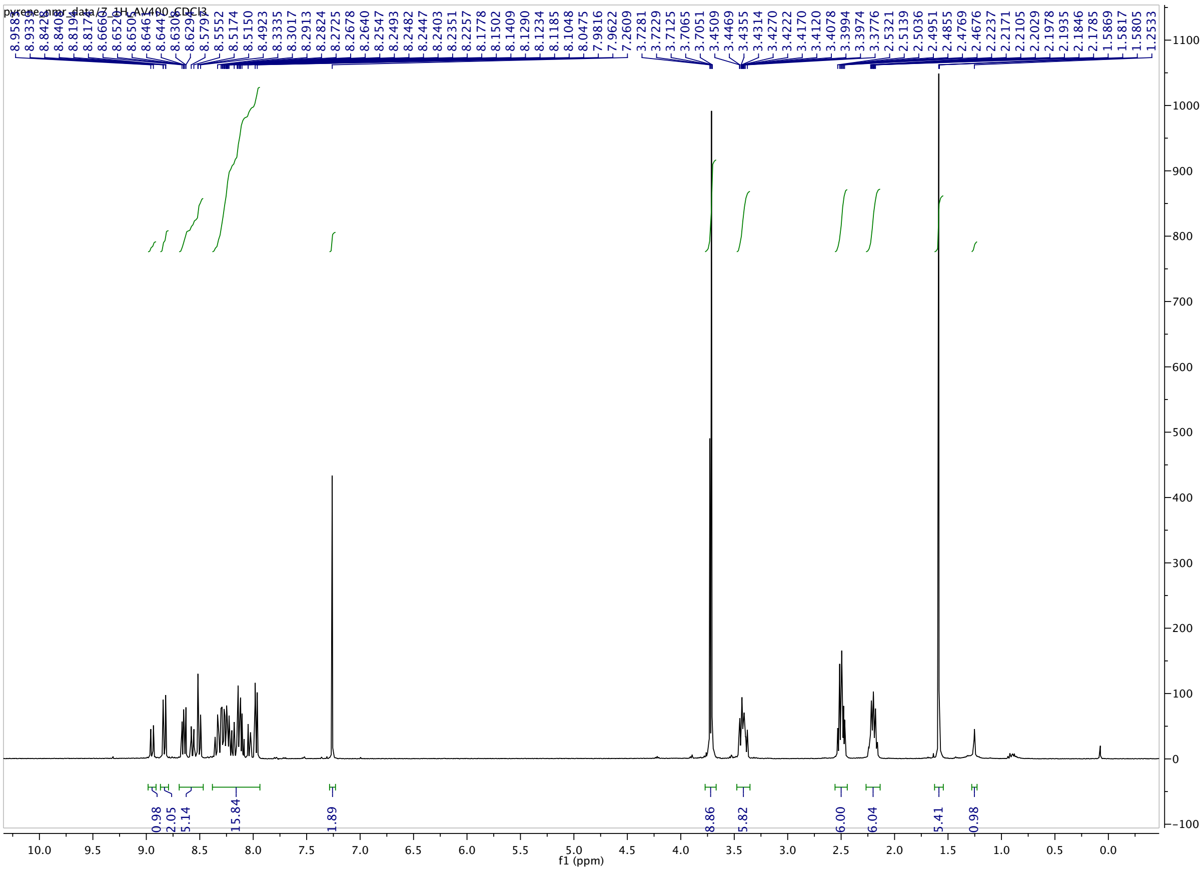

^{1}H NMR spectrum of nitropyrene **10** (400 MHz, $CDCl_3$).

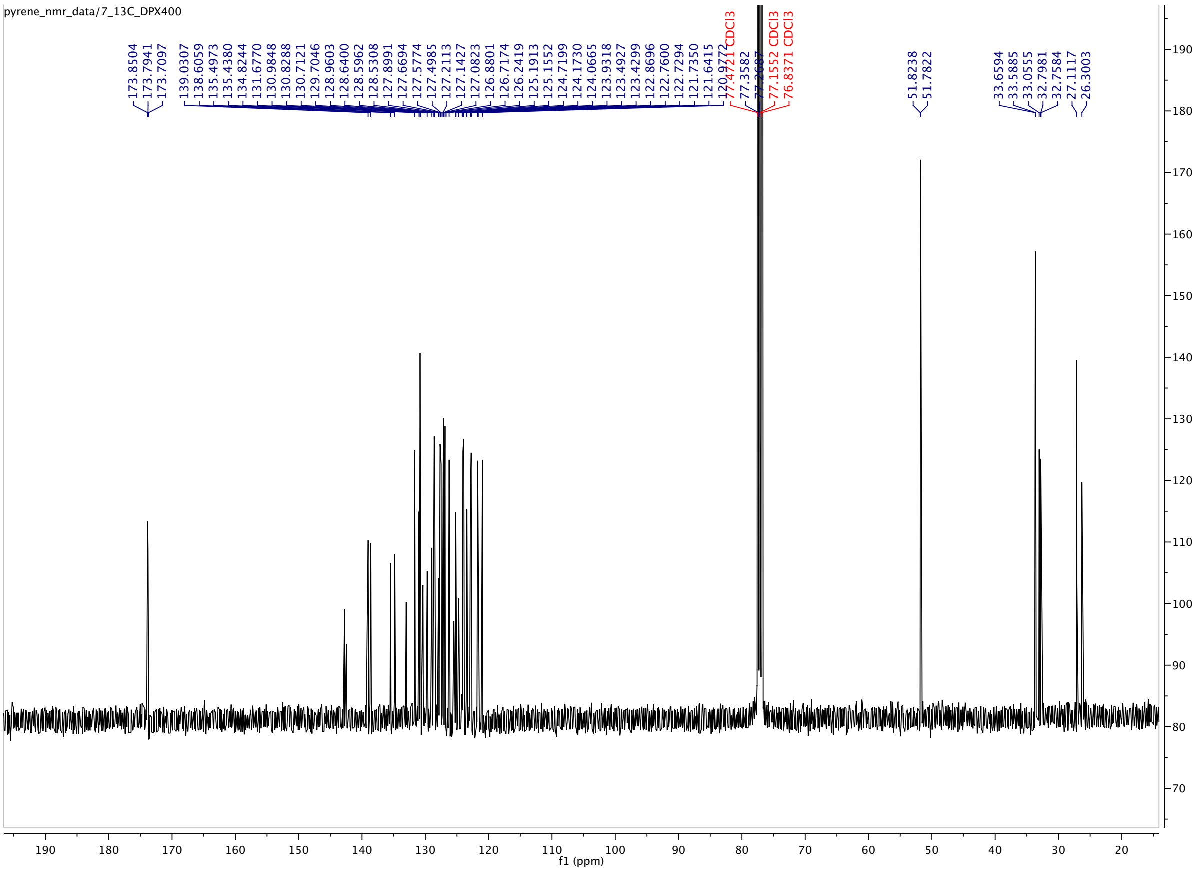

^{13}C NMR spectrum of nitropyrene **10** (100 MHz, $CDCl_3$).

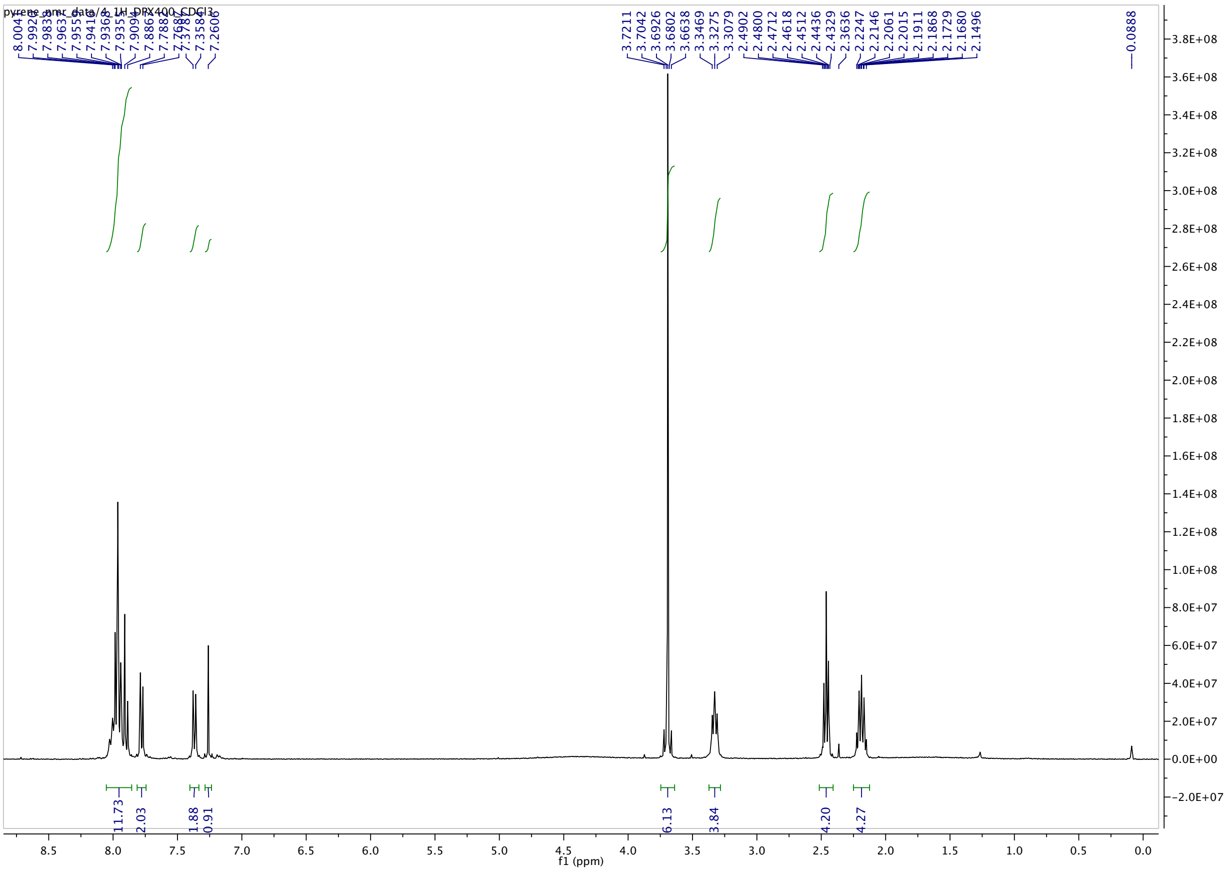

^{1}H NMR spectrum of 6-aminopyrene **2** (400 MHz, $CDCl_3$).

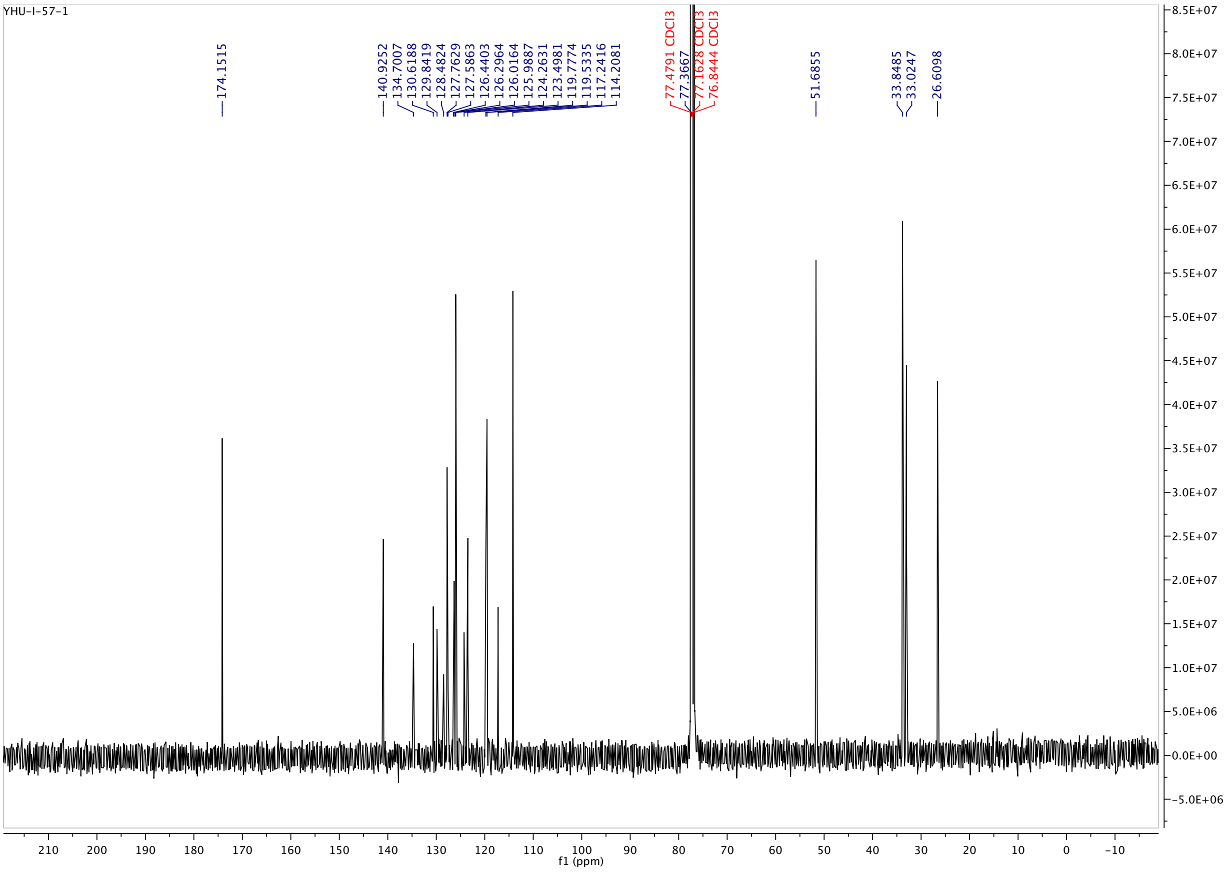

^{13}C NMR spectrum of 6-aminopyrene **2** (100 MHz, $CDCl_3$).

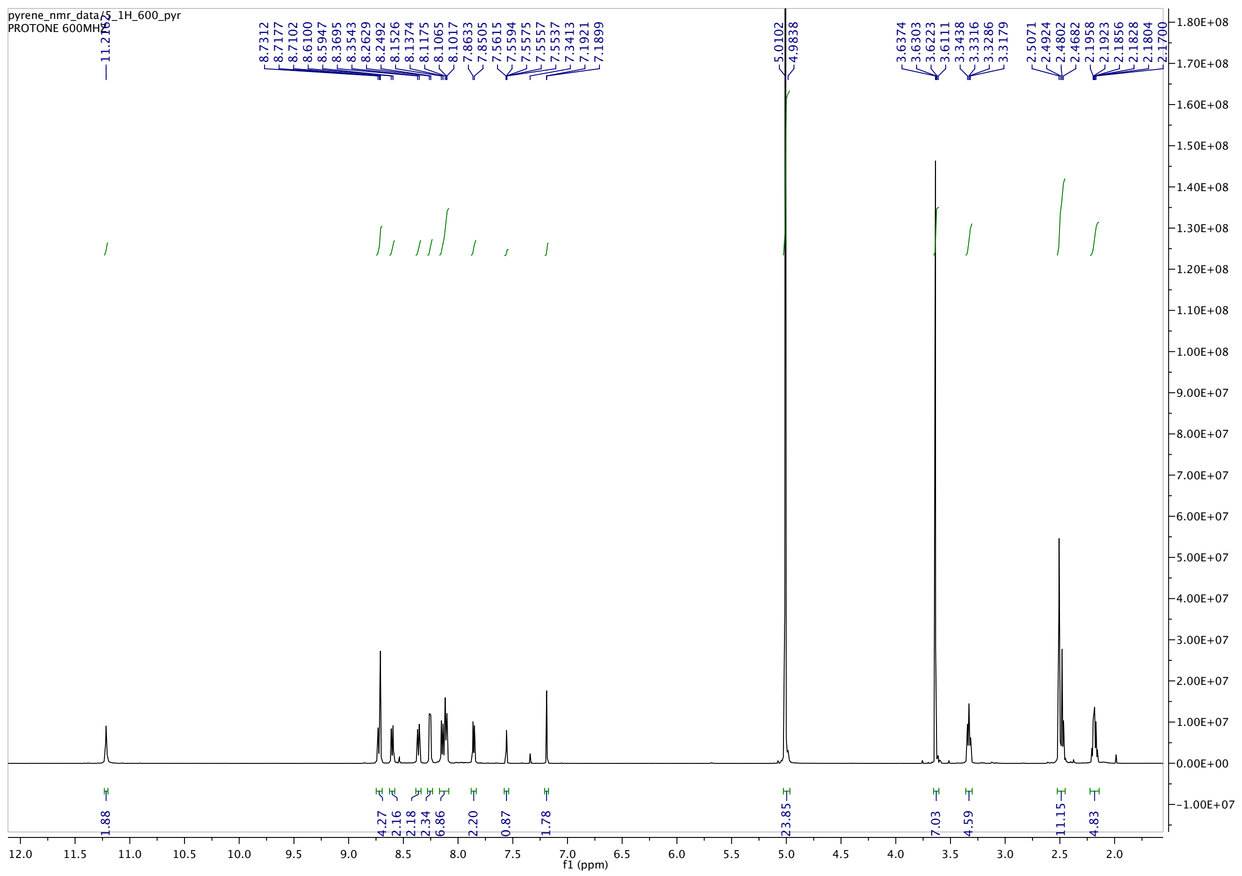

^{1}H NMR spectrum of 6-amidopyrene **3** (600 MHz, pyridine-d_5).

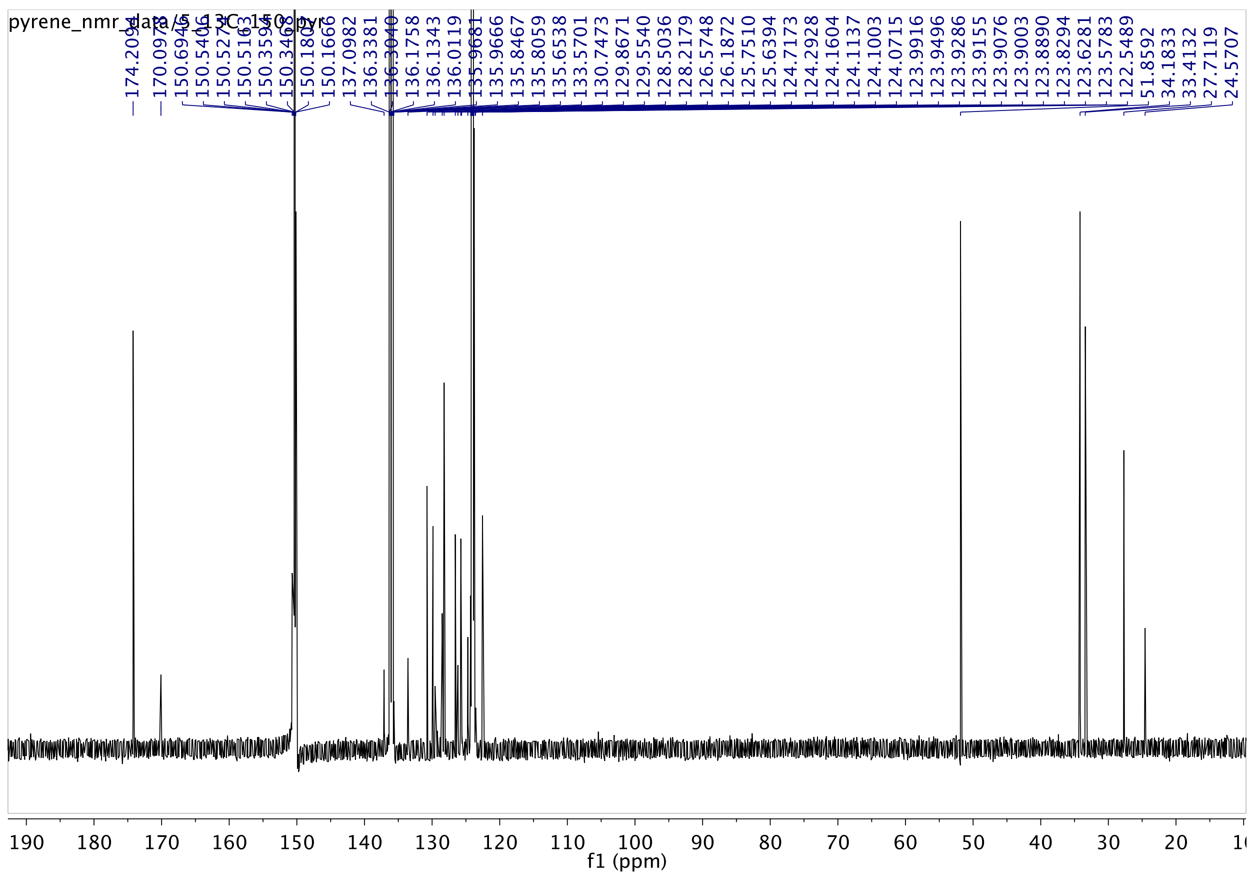

^{13}C NMR spectrum of 6-amidopyrene **3** (150 MHz, pyridine-d_5).

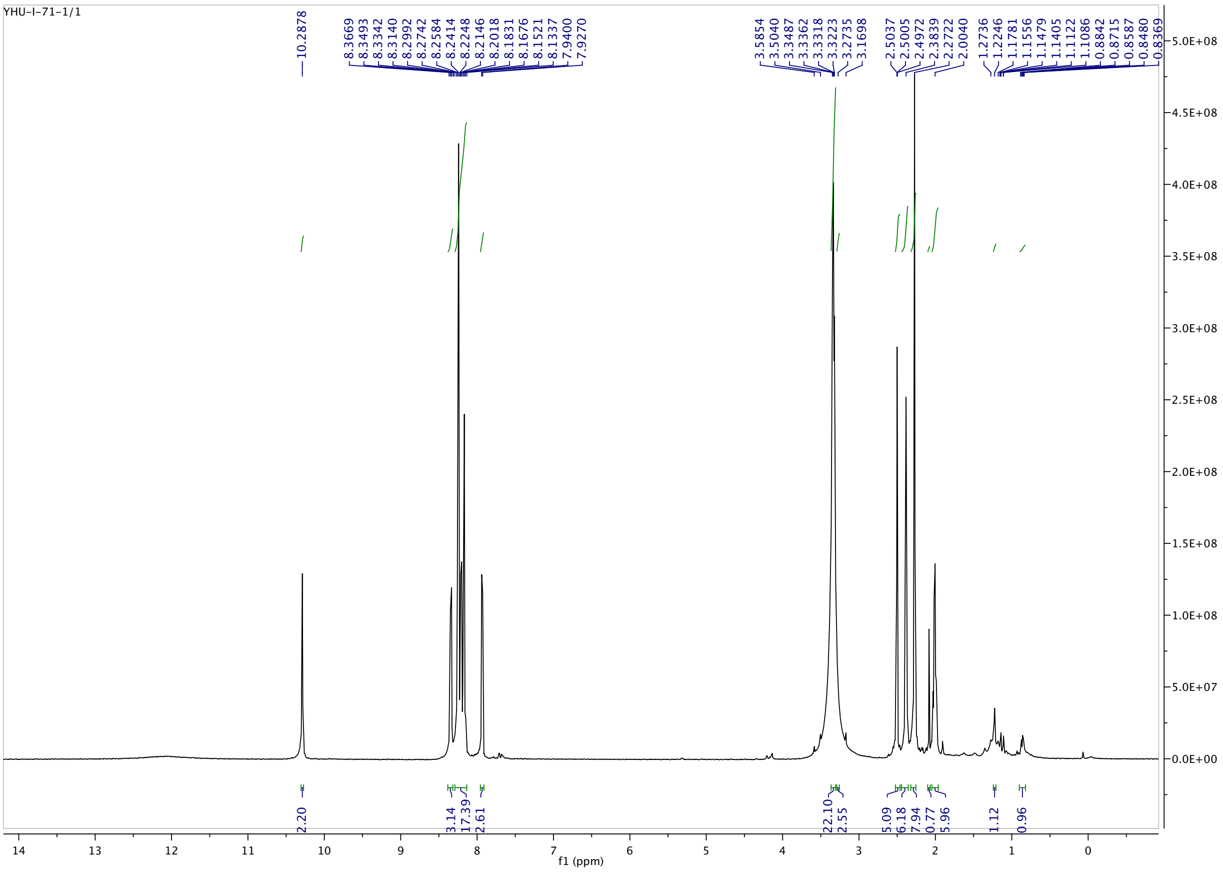

^{1}H NMR spectrum of carboxylic acid **11** (600 MHz, DMSO-d_6).

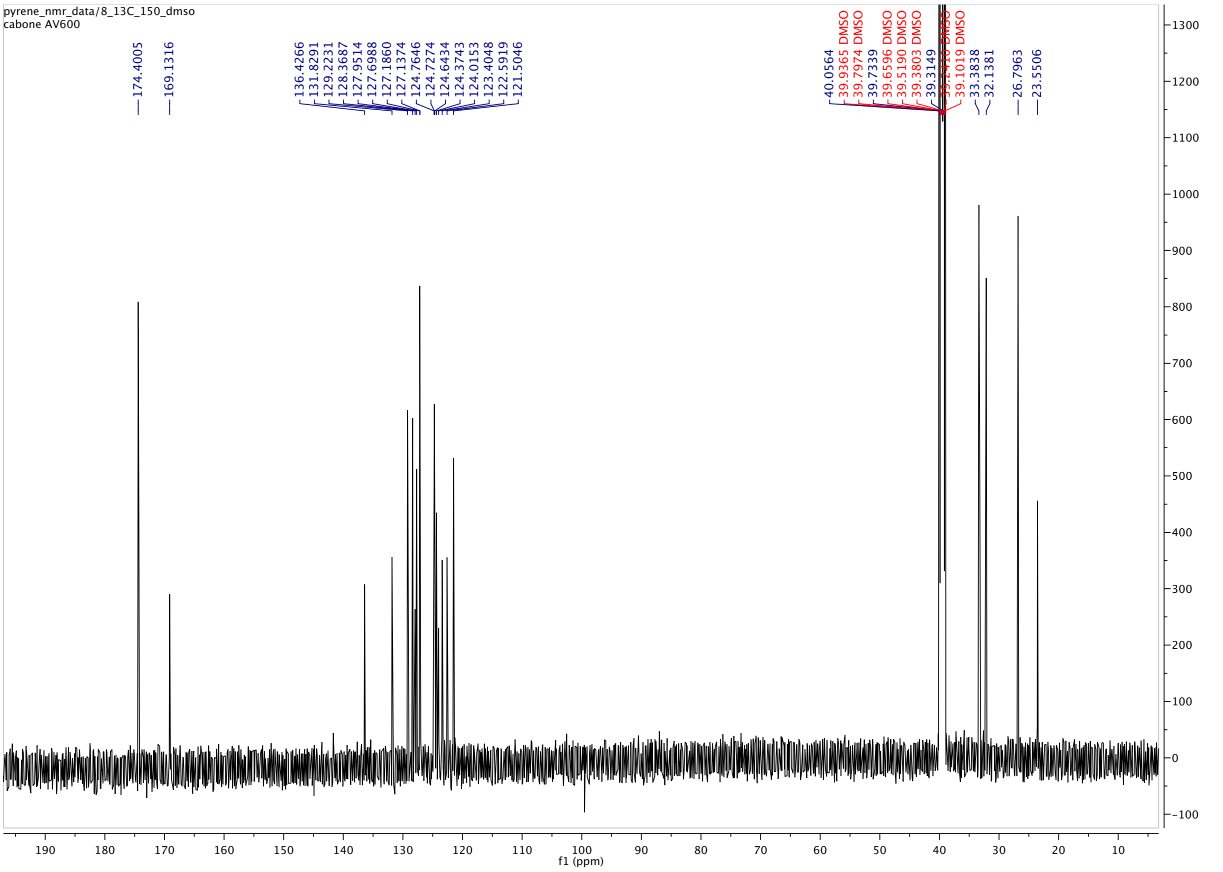

^{13}C NMR spectrum of carboxylic acid **11** (150 MHz, DMSO-d_6).

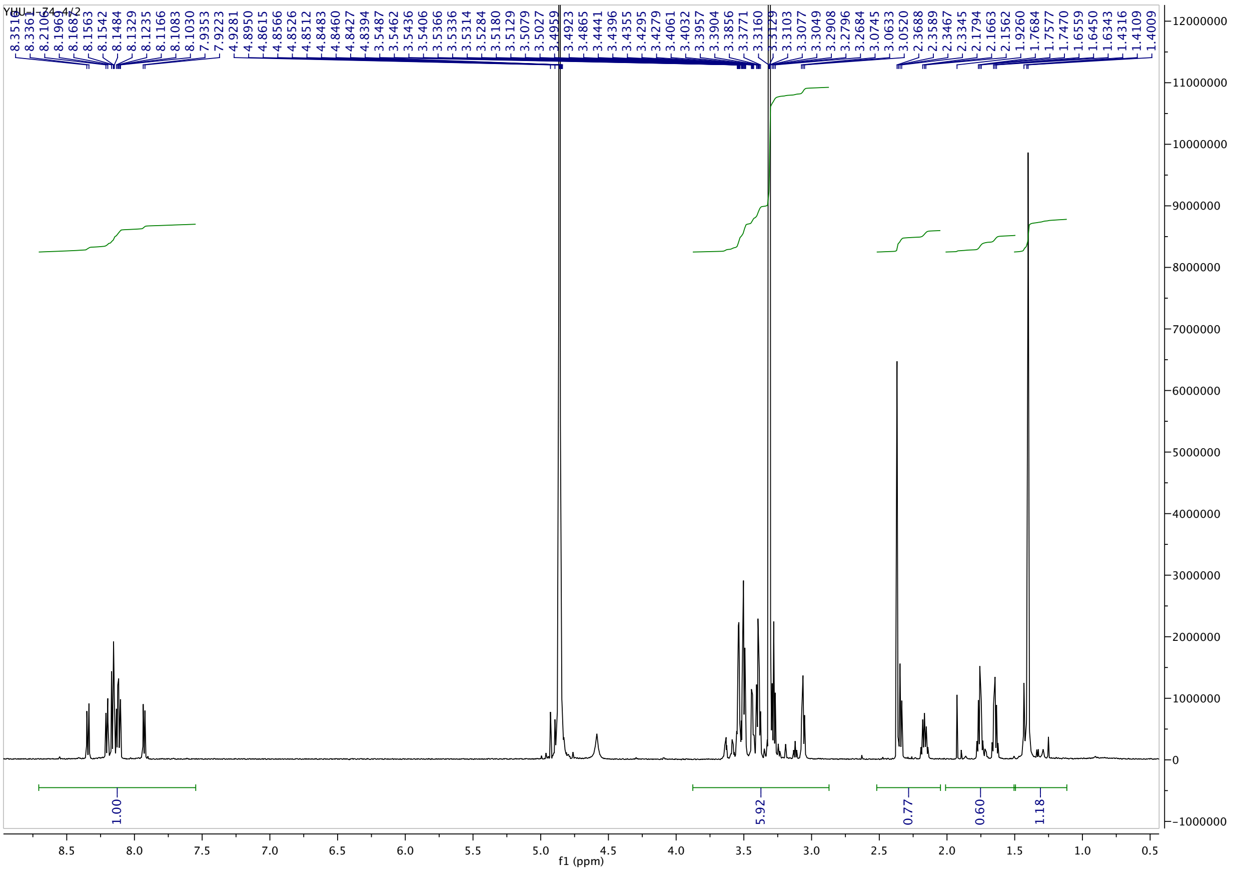

^{1}H NMR spectrum of amide **13** (600 MHz, CD_3OD).

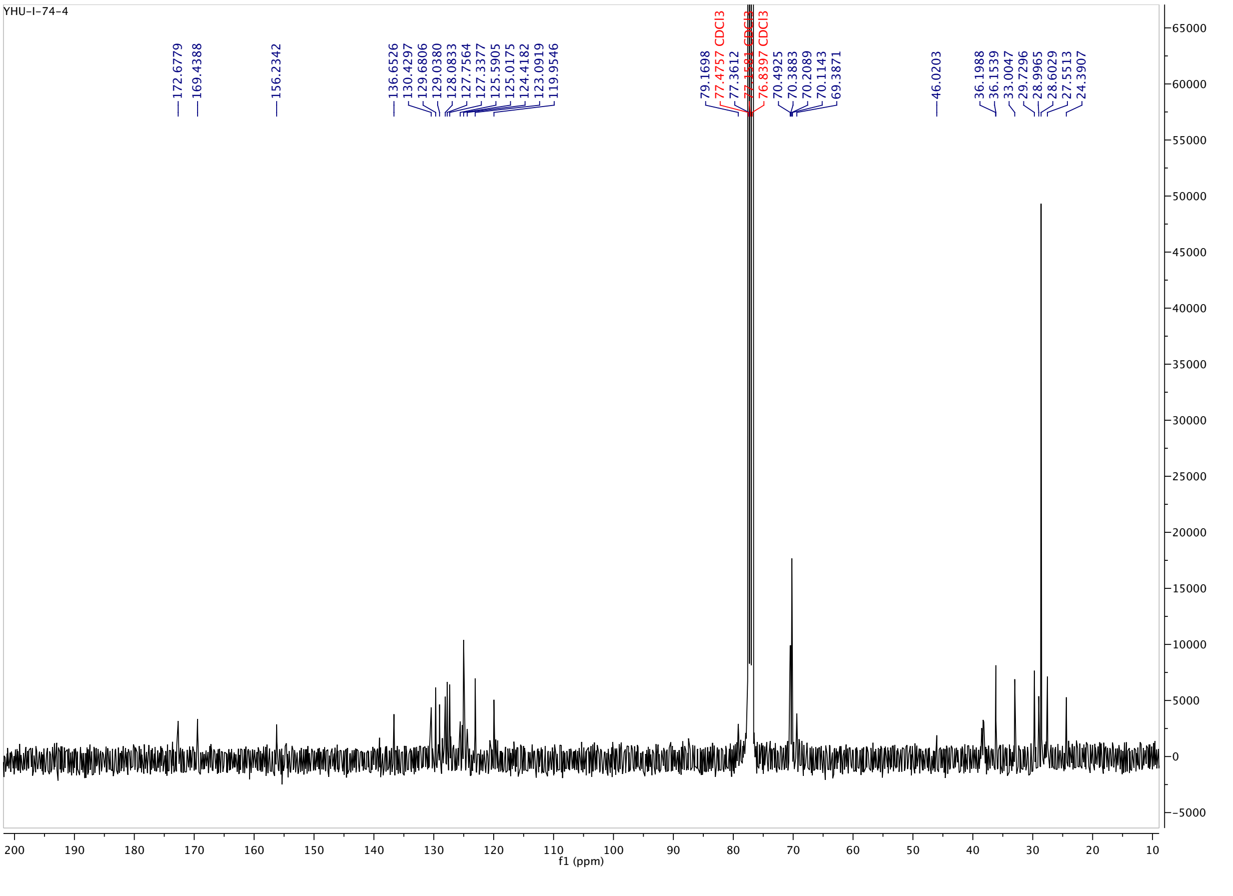

^{13}C NMR spectrum of amide **13** (100 MHz, $CDCl_3$).

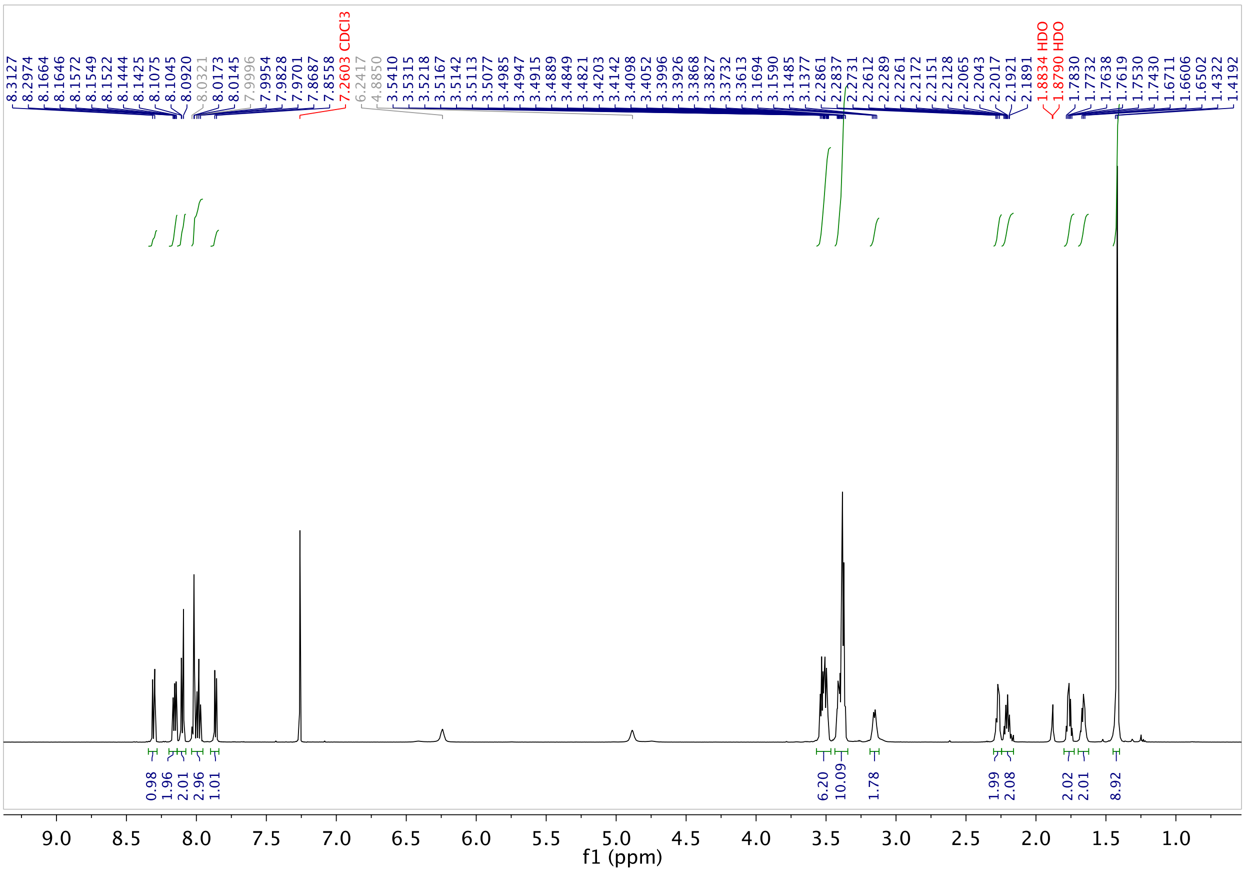

^{1}H NMR spectrum of amide **14** (600 MHz, $CDCl_3$).

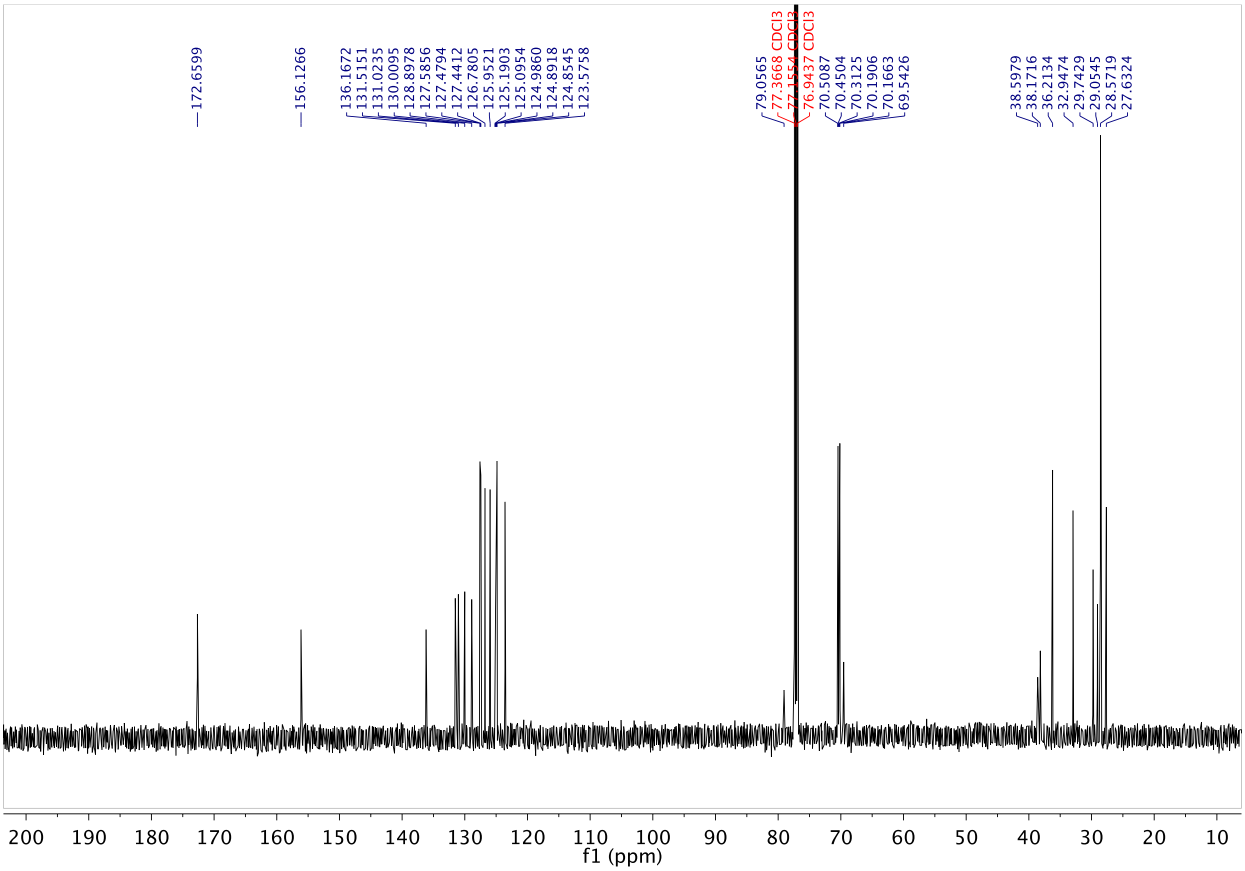

^{13}C NMR spectrum of amide **14** (150 MHz, $CDCl_3$).

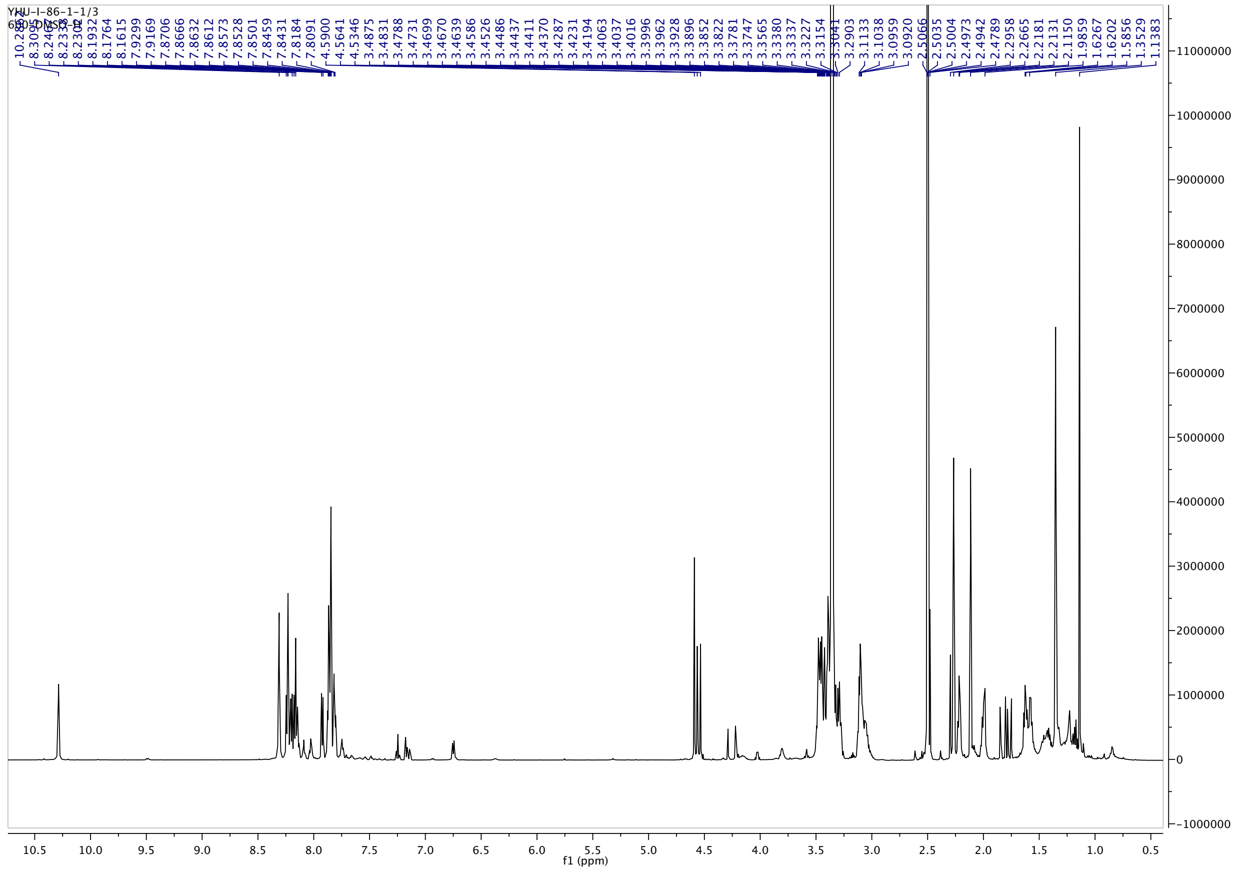

^{1}H NMR spectrum of lysine amide **16** (600 MHz, DMSO-d_6).

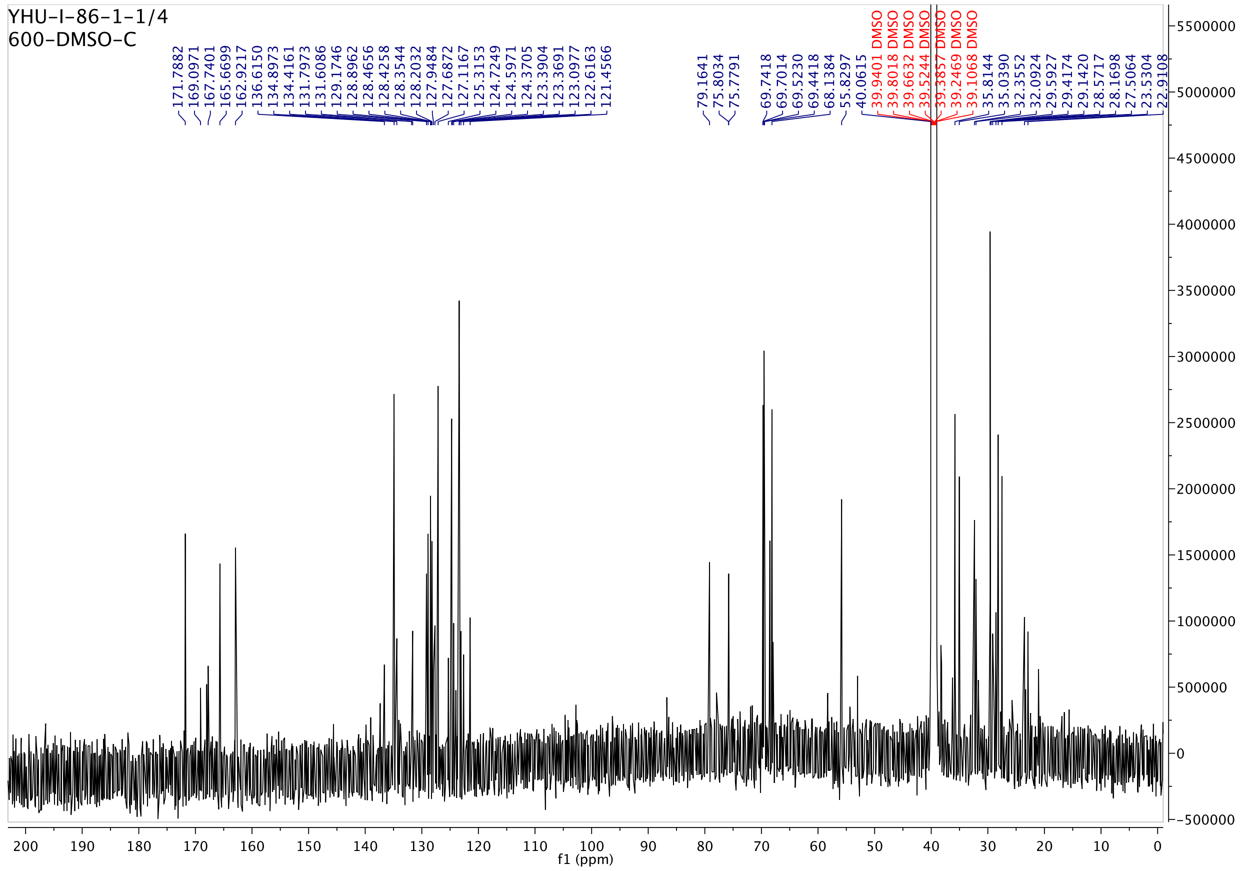

^{13}C NMR spectrum of lysine amide **16** (150 MHz, DMSO-d_6).

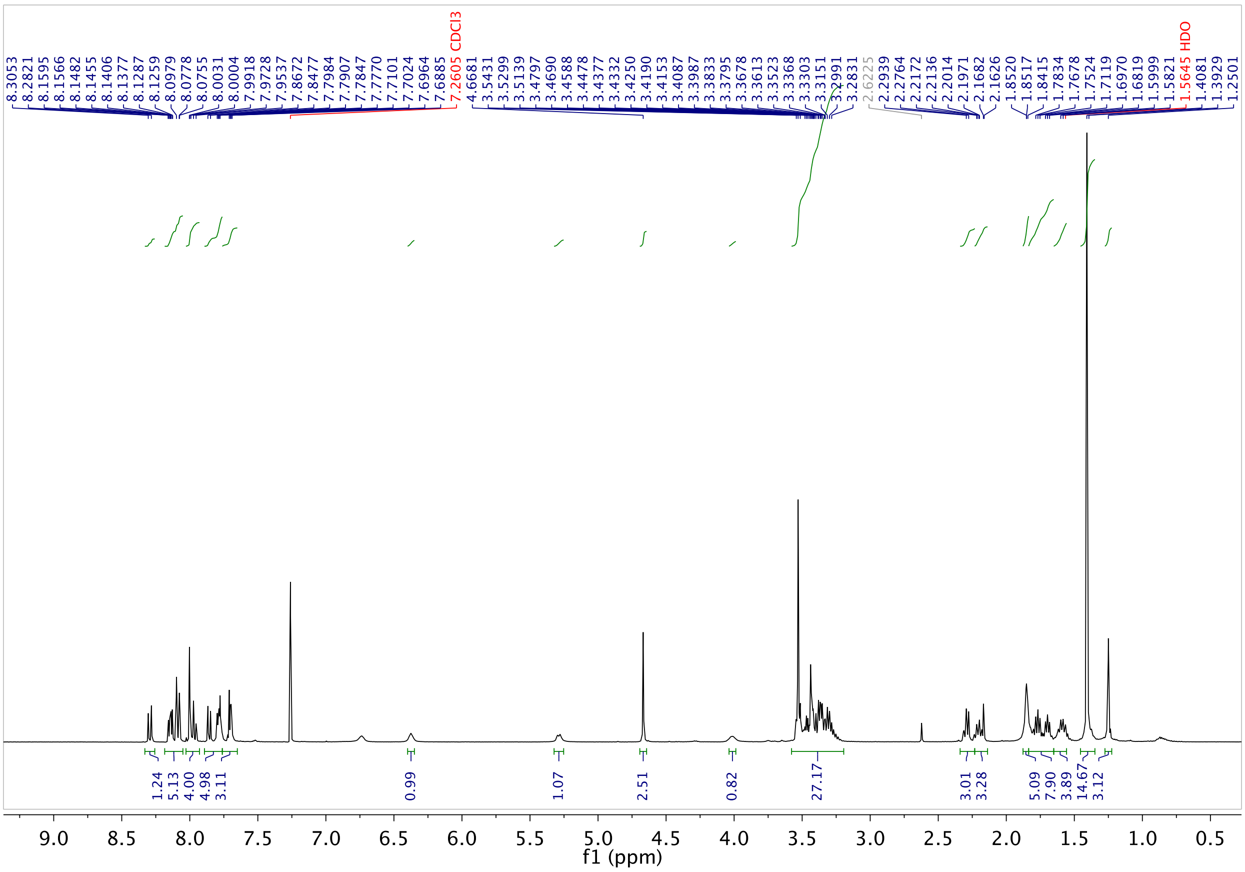

^{1}H NMR spectrum of lysine amide **17** (400 MHz, $CDCl_3$).

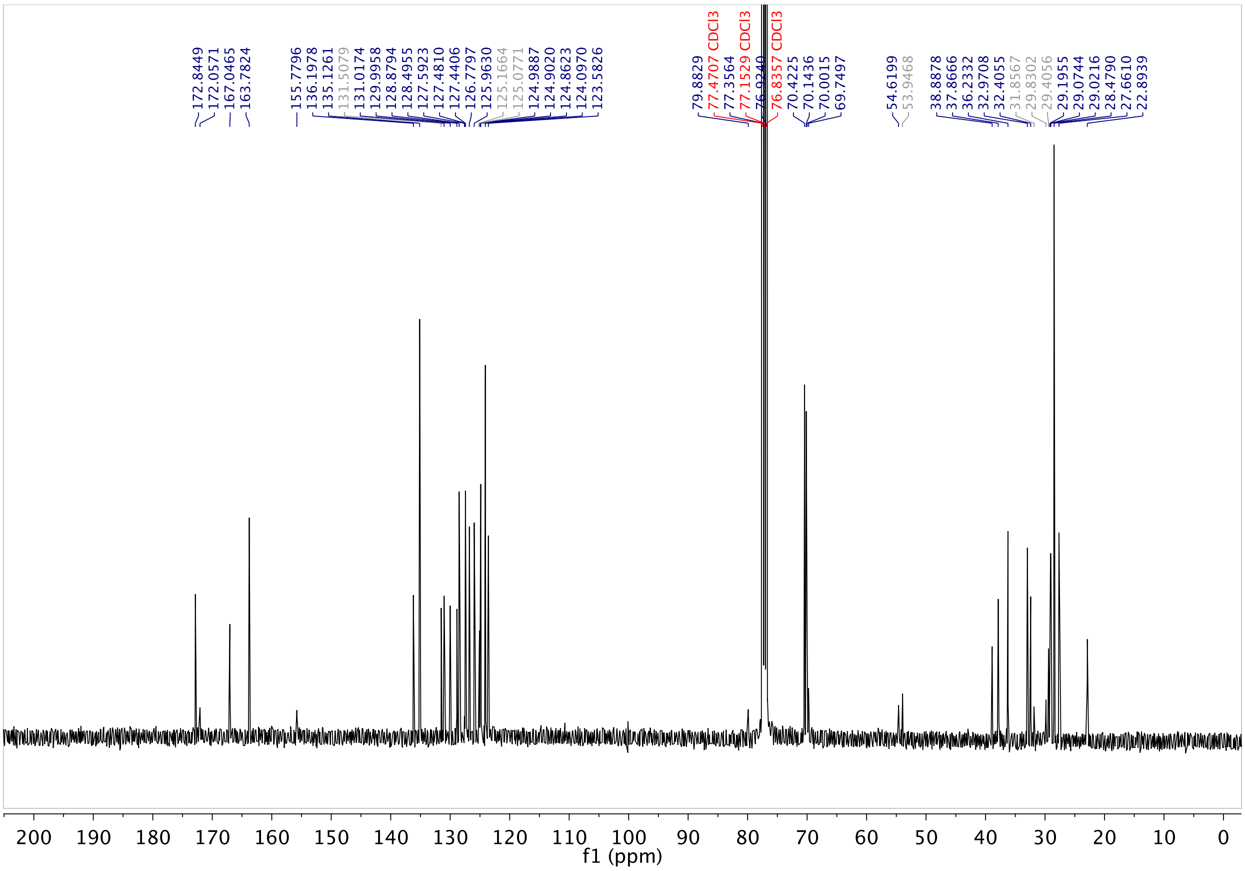

^{13}C NMR spectrum of lysine amide **17** (100 MHz, $CDCl_3$).

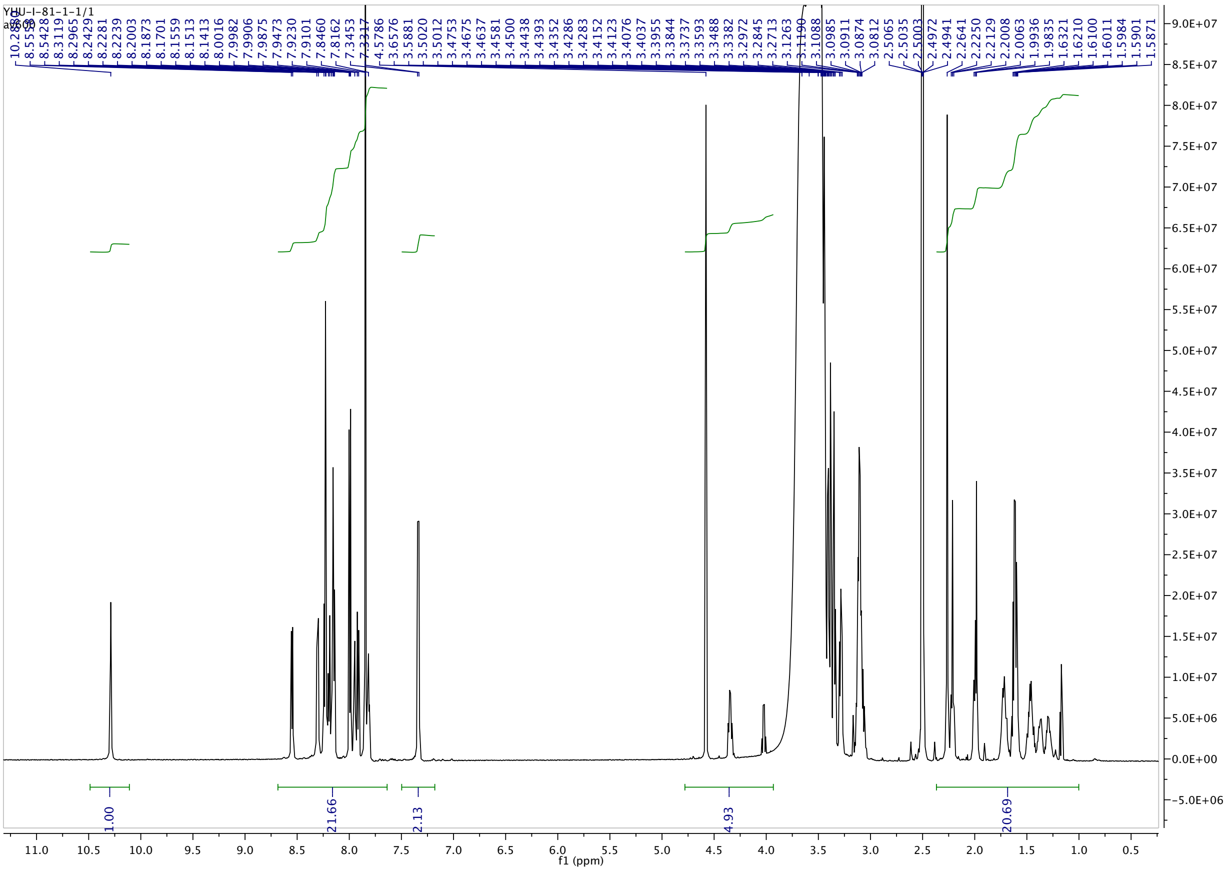

^{1}H NMR spectrum of diazirine amide **19** (600 MHz, DMSO-d_6).

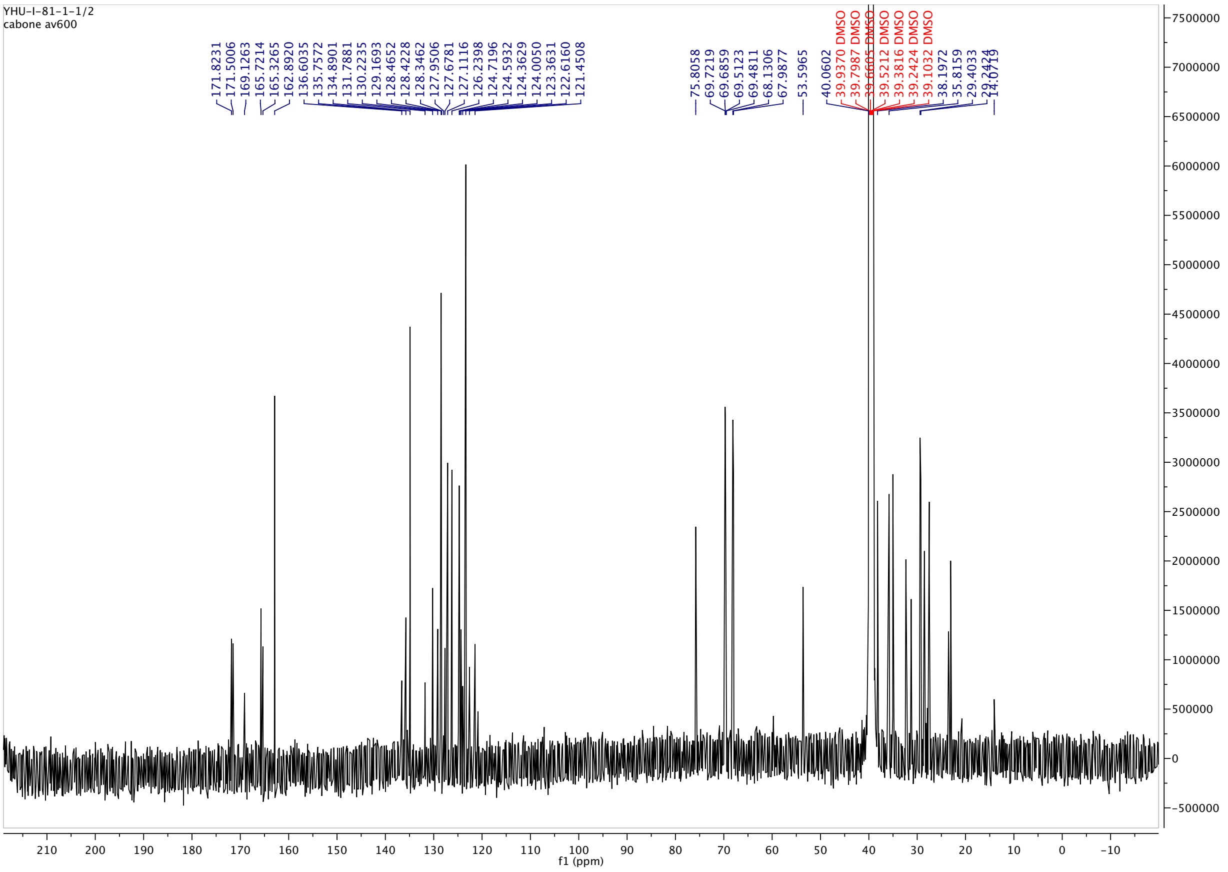

^{13}C NMR spectrum of diazirine amide **19** (150 MHz, DMSO-d_6).

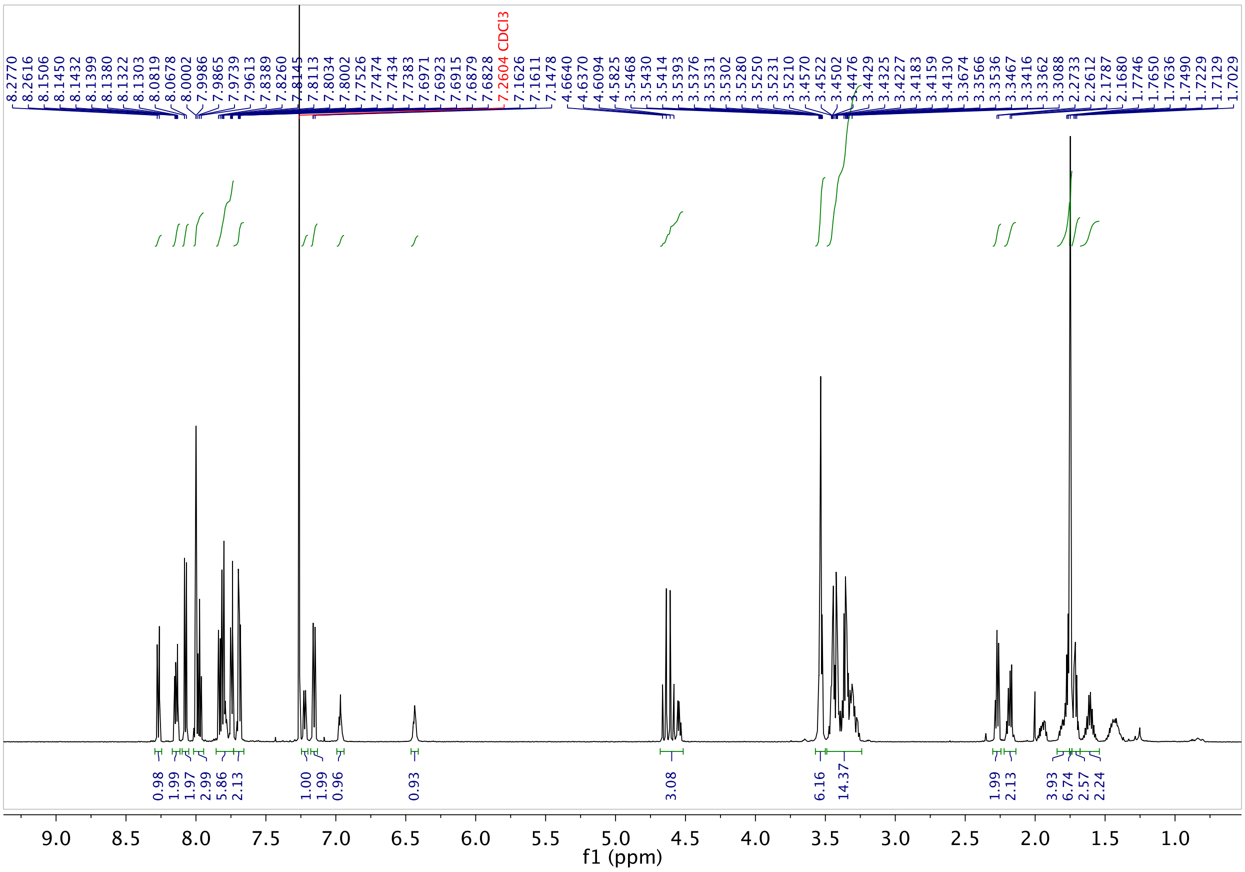

^{1}H NMR spectrum of diazirine amide **20** (600 MHz, $CDCl_3$).

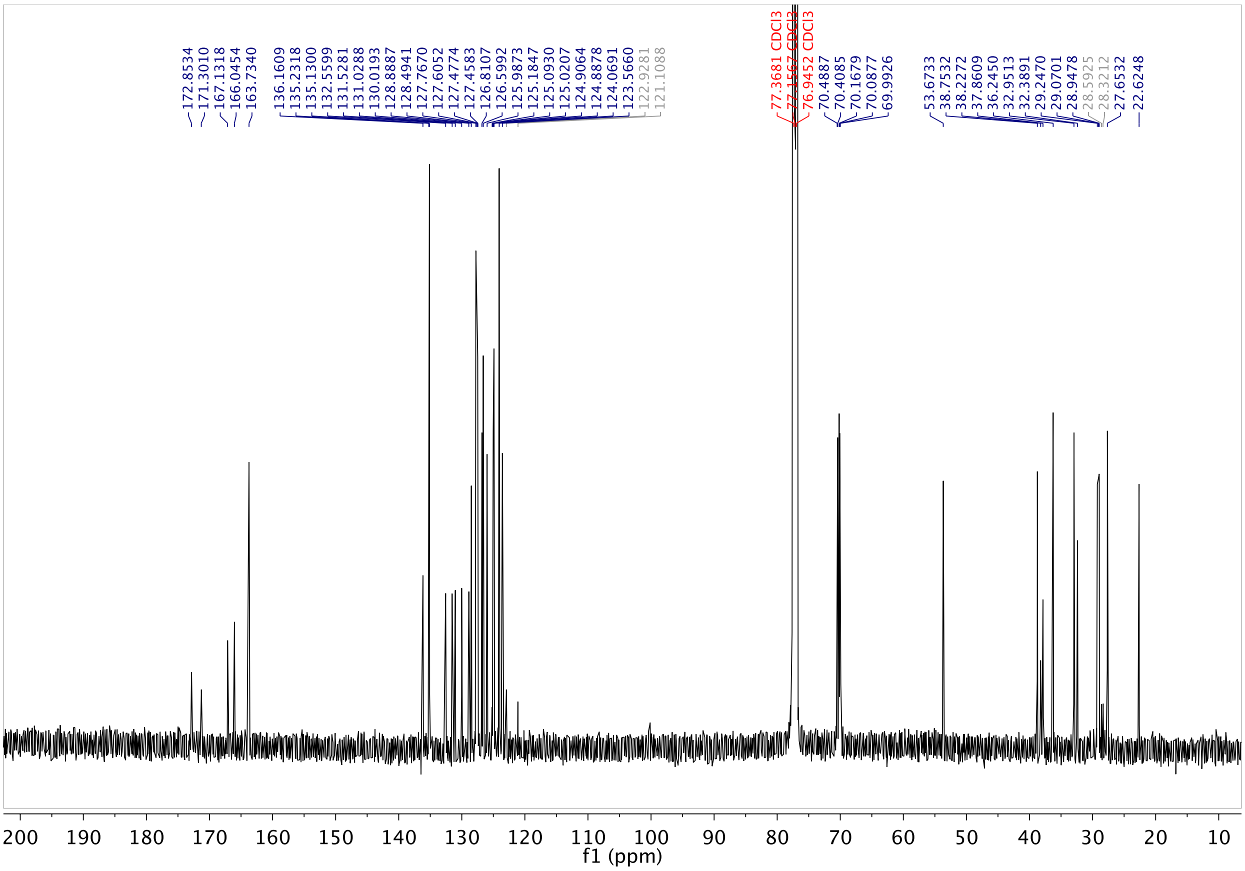

^{13}C NMR spectrum of diazirine amide **20** (150 MHz, $CDCl_3$).

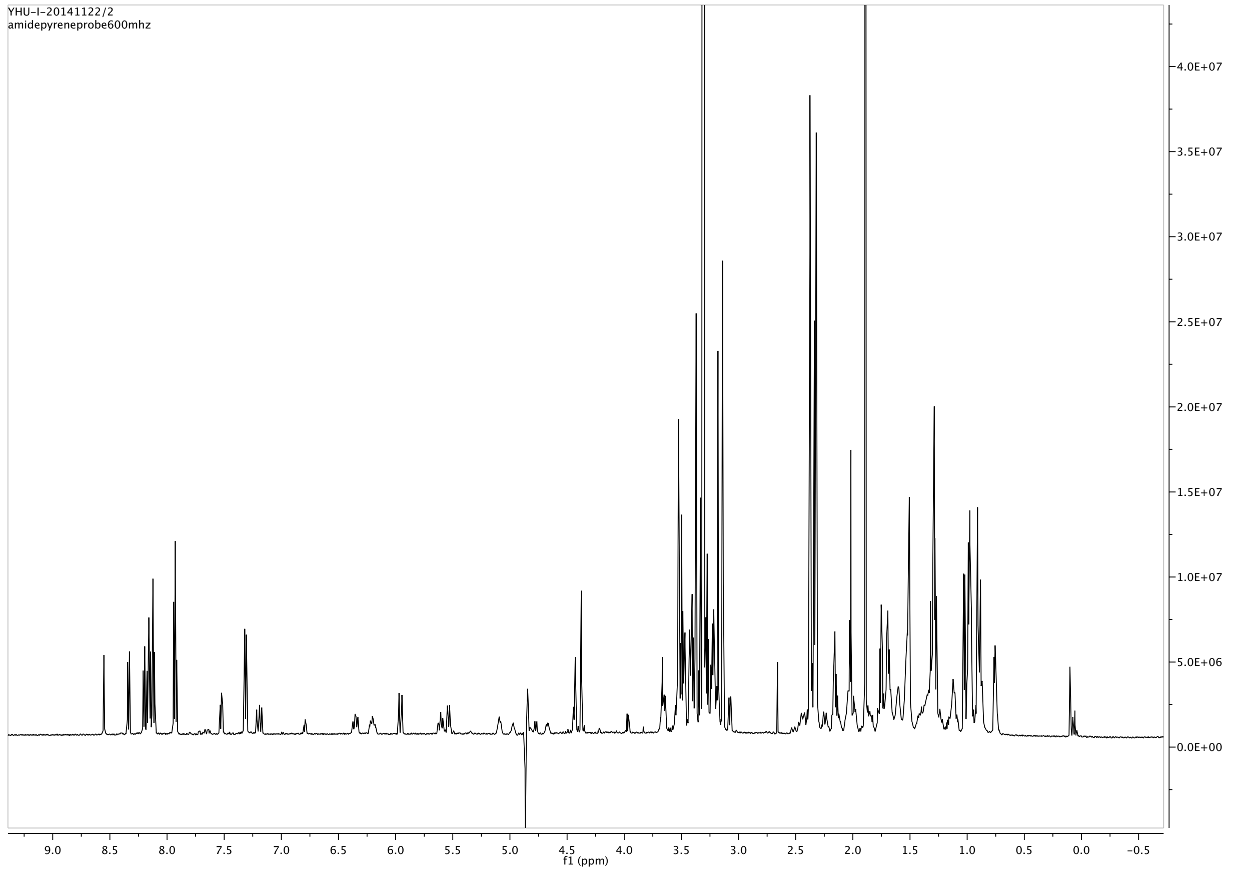

^{1}H NMR spectrum of ApA–PaP (**4**) (600 MHz, CD_3OD).

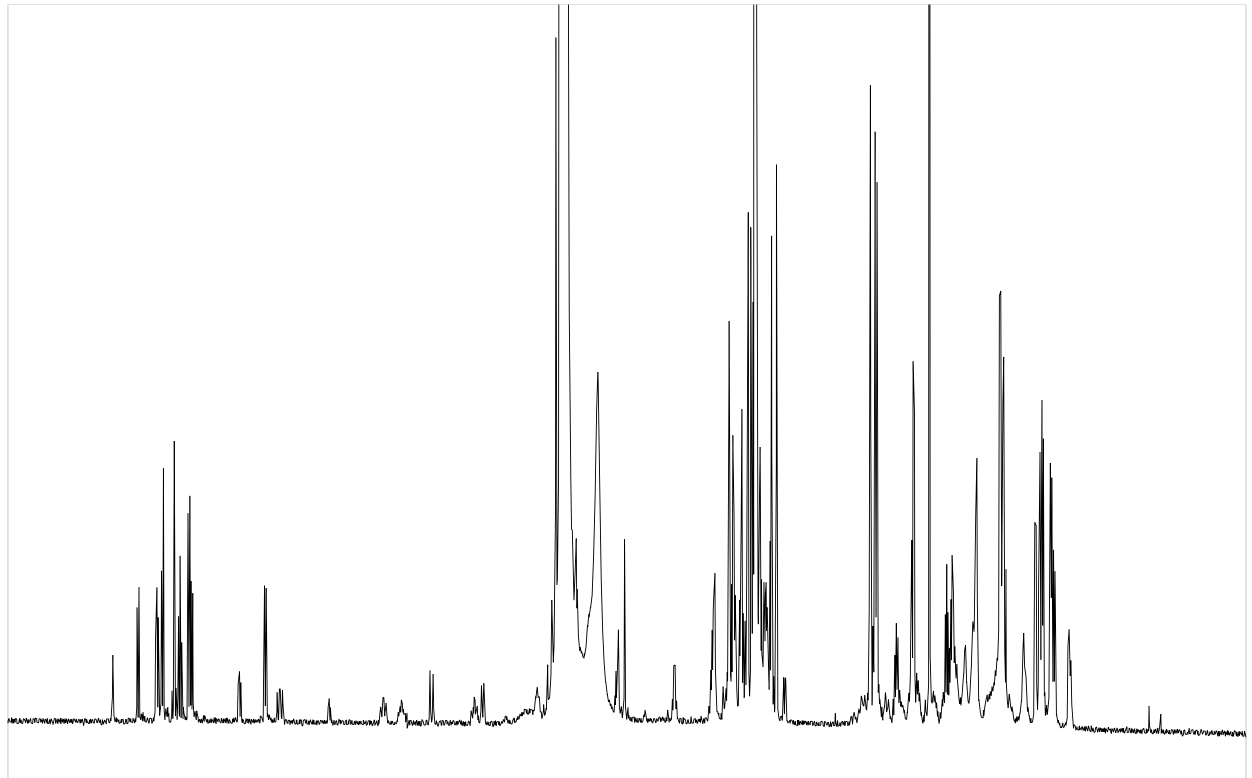

^{1}H NMR spectrum of ApA–PP (**5**) (600 MHz, CD_3OD).

HPLC charts

ApA–PaP (**4**)

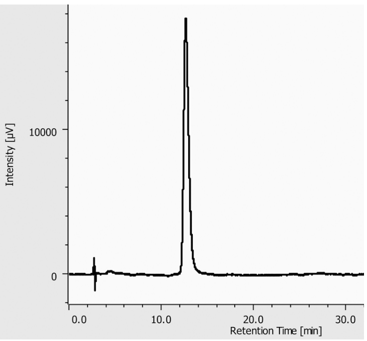

HPLC conditions: Column, Develosil ODS-HG-5 (ϕ 4.6 × 250 mm); Eluate, MeOH / 20 mM NH_4OAc = 83/17; Detection, UV254 nm; Flow rate, 1 mL/min. Stereoisomers for the C34 oxime moiety in **4** were not separable. t_R = 12.7 min.

ApA–PP (**5**)

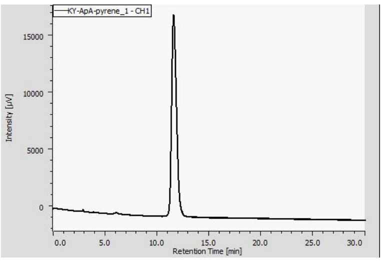

HPLC conditions: Column, Develosil ODS-HG-5 (ϕ 4.6 × 250 mm); Eluate, MeOH / 20 mM NH_4OAc = 90/10; Detection, UV254 nm; Flow rate, 1 mL/min. Stereoisomers for the C34 oxime moiety in **5** were not separable. t_R = 11.6 min.

MeOH-adduct of ApA–PaP (**6**)

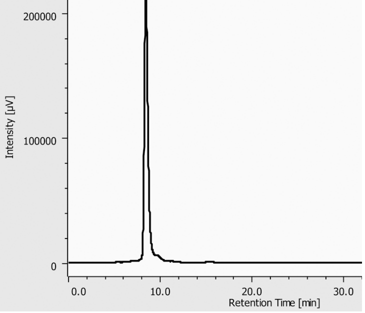

HPLC conditions: Column, Develosil ODS-HG-5 (ϕ 4.6 × 250 mm); Eluate, MeOH / 20 mM NH_4OAc = 83/17; Detection, fluorescence λ_{ex} 337 nm and λ_{em} 409 nm; Flow rate, 1 mL/min. Stereoisomers for the C34 oxime moiety in **6** were not separable. t_R = 8.3 min.

MeOH-adduct of ApA–PP (**7**)

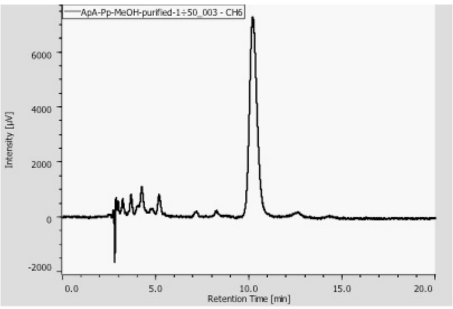

HPLC conditions: Column, Develosil ODS-HG-5 (ϕ 4.6 × 250 mm); Eluate, MeOH / 20 mM NH_4OAc = 88/12; Detection, UV254 nm; Flow rate, 1 mL/min. Stereoisomers for the C34 oxime moiety in **7** were not separable. t_R = 10.2 min.

Water-adduct of ApA–PaP (**8**)

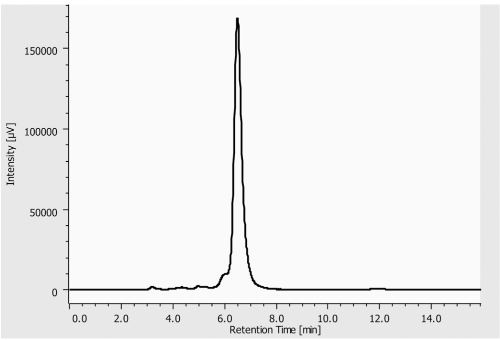

HPLC conditions: Column, Develosil ODS-HG-5 (ϕ 4.6 × 250 mm); Eluate, MeOH / 20 mM NH_4OAc = 83/17; Detection, fluorescence λ_{ex} 337 nm and λ_{em} 409 nm; Flow rate, 1 mL/min. Stereoisomers for the C34 oxime moiety in **8** were not separable. t_R = 6.4 min.
